# Tuning MOF/polymer interfacial pore geometry in mixed matrix membrane for upgrading CO_2_ separation performance

**DOI:** 10.1126/sciadv.adk5846

**Published:** 2024-07-10

**Authors:** Aydin Ozcan, Dong Fan, Shuvo Jit Datta, Alejandro Diaz-Marquez, Rocio Semino, Youdong Cheng, Biplab Joarder, Mohamed Eddaoudi, Guillaume Maurin

**Affiliations:** ^1^ ICGM, University of Montpellier, CNRS, ENSCM, Montpellier, France.; ^2^ Materials Technologies, TÜBITAK Marmara Research Center, 41470 Gebze, Kocaeli, Türkiye.; ^3^ School of Materials Science and Engineering, Chongqing Jiaotong University, Chongqing 400074, P.R. China.; ^4^ Division of Physical Science and Engineering (PSE), Advanced Membrane and Porous Materials Center, King Abdullah University of Science and Technology (KAUST), Thuwal 23955-6900, Saudi Arabia.; ^5^ Division of Physical Science and Engineering, Advanced Membrane and Porous Materials Center, Functional Materials Design, Discovery and Development (FMD3), KAUST, Thuwal 23955-6900, Saudi Arabia.; ^6^ CNRS, Physico-chimie des Electrolytes et Nanosystèmes Interfaciaux, PHENIX, Sorbonne Université, F-75005 Paris, France.

## Abstract

The current paradigm considers the control of the MOF/polymer interface mostly for achieving a good compatibility between the two components to ensure the fabrication of continuous mixed-matrix metal-organic framework (MMMOF) membranes. Here, we unravel that the interfacial pore shape nanostructure plays a key role for an optimum molecular transport. The prototypical ultrasmall pore AlFFIVE-1-Ni MOF was assembled with the polymer PIM-1 to design a composite with gradually expanding pore from the MOF entrance to the MOF/polymer interfacial region. Concentration gradient–driven molecular dynamics simulations demonstrated that this pore nanostructuring enables an optimum guided path for the gas molecules at the MOF/polymer interface that decisively leads to an acceleration of the molecular transport all along the MMMOF membrane. This numerical prediction resulted in the successful fabrication of a [001]-oriented nanosheets AlFFIVE-1-Ni/PIM-1 MMMOF membrane exhibiting an excellent CO_2_ permeability, better than many MMMs, and ideally associated with a sufficiently high CO_2_/CH_4_ selectivity that makes this membrane very promising for natural gas/biogas purification.

## Teaser

Engineering of interfacial MOF/polymer pore nanostructuring for the development of advanced CO_2_ selective mixed matrix membranes.

## INTRODUCTION

The energy needs for sorting or purifying key substances, e.g., industrial gases, fine chemicals, and fresh water, represents a tremendous amount of annual global energy consumption. The development of more energy-efficient and environmentally friendly separation technologies is therefore a major societal challenge that has stimulated a massive academia industry effort over the last decades (*1*–*3*). In this regard, membrane technology has gained huge momentum as a prominent alternative to energy-intensive separation processes, including distillation among others (*4*, *5*). However, the attractiveness of the first-generation polymeric membranes has been limited by their tradeoff between permeability, i.e., how fast molecules pass through the membranes, and selectivity, i.e., the extent to which the desirable molecules are separated from the others, called Robeson upper bound (*6*). Mixed matrix membranes (MMMs), an appealing combination of easily processable polymers and highly selective ordered porous materials, i.e., zeolites (*7*), metal-organic frameworks (MOFs) (*8*–*13*), covalent-organic frameworks (*14*), or carbon nanotubes (*15*) have been envisioned as next-generation high-performance membranes for overcoming this permeability-selectivity tradeoff.

Distinctly, ultrasmall pore MOFs and zeolites display remarkable performance as molecular separating agents, notably owing to their uniform porosity and/or the chemical functionality decorating their pore walls (*16*–*18*). MMMs integrating MOFs (MMMOF membranes) [MIL-53(Al)-NH_2_] (*9*), Cu-BDC (*19*), co-benzenedicarboxylate (*20*), AlFFIVE-1-Ni (*21*), or zeolites (4A) (*22*) into a polymer matrix have shown promising separation performance for a range of gas mixtures however associated with far from optimal gas permeability (*23*, *24*). This overall observation calls for the design of sweet-pot MMMOF membranes encompassing high permeability (productivity) and reasonably high selectivity (efficiency), to make a leap forward in the field toward a future promotion of this class of membranes at the market.

**Fig. 1.**
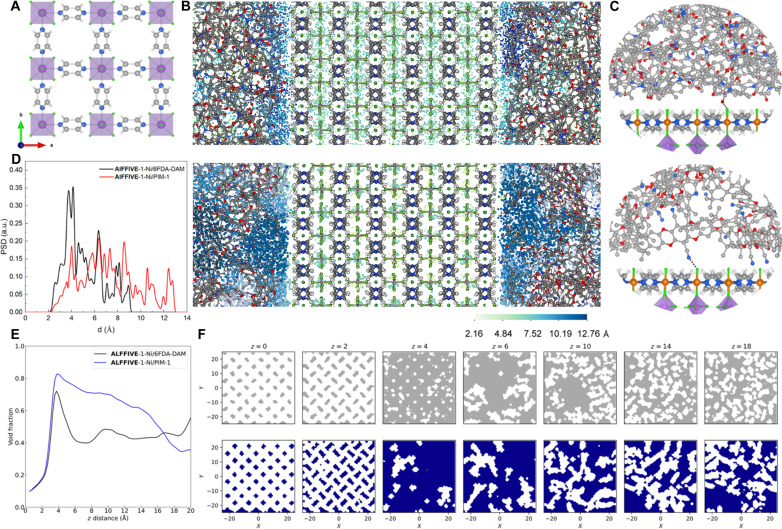
In silico construction and analysis of the MOF/polymer composite models. (**A**) Top view of the crystal structure of AlFFIVE-1-Ni. F, N, C, Ni, Al, and H are shown in green, blue, gray, orange, purple, and white, respectively. (**B**) AlFFIVE-1-Ni/6FDA-DAM (top) and AlFFIVE-1-Ni/PIM-1 atomistic (bottom) models with the corresponding 3D pore size. The pore diameter (in Å) is colored according to the legend. (**C**) Illustration of the polymer organization at the vicinity of the MOF surface AlFFIVE-1-Ni/6FDA-DAM (top) and AlFFIVE-1-Ni/PIM-1 (bottom). Black dashed lines represent the preferential interactions between atoms of the polymer and the MOF. (**D**) Calculated pore size distributions (PSD) for the two MMMOF membrane models. The PSD calculation of the composite excludes the contribution of the AlFFIVE-1-Ni. a.u., arbitrary units. (**E**) Void fraction for the two composite models of (B) plotted along the *z* direction within a 20-Å distance away from the reference Ni position (*z* = 0) of the first layer of AlFFIVE-1-Ni (z is the direction normal to the MOF surface slab model). (**F**) Representative illustration of the free porosity over the *xy* plane for AlFFIVE-1-Ni/6FDA-DAM (top) and AlFFIVE-1-Ni/PIM-1 (bottom) for different *z* values when one moves away from the MOF surface, where colored regions (gray and blue, respectively) represent void spaces, and white regions denote the presence of polymers. The full set of data for the overall *z* distances are reported in fig. S8 and S10, respectively.

In this quest, combining a high-selective ultrasmall pore one-dimensional (1D) channel MOF with a highly permeable polymer offers a unique opportunity to engineer an optimum MMMOF membrane to achieve highly efficient molecular separations. As a proof-of-concept, we opted for AlFFIVE-1-Ni (KAUST-8) made of square two-dimensional layers of Ni^2+^ connected to pyrazine linkers and pillared by [AlF_5_(H_2_O)]^2−^ anions in the third dimension, as a prototypical ultrasmall pore 1D channel MOF (Fig. 1A). Beyond its outstanding properties for gas dehydration (*25*) and acid gas removal (*26*, *27*), this MOF has been recently used as filler into the soft and low-permeable polymeric 6FDA-DAM matrix resulting into to a MMM that showed a high CO_2_/CH_4_ selectivity accompanied by a CO_2_ permeability that still needs to be improved (*21*). We therefore deliberately aimed to associate AlFFIVE-1-Ni with the more rigid and highly permeable polymer of intrinsic microporosity (PIM-1) to design a MMMOF membrane with enhanced permeability whilst retaining relatively high selectivity.

The targeted AlFFIVE-1-Ni/PIM-1 composite was first created in silico using a computational strategy combining quantum and equilibrium force field molecular dynamics (E-MD) simulations. The interfacial MOF/polymer organization was scrutinized to first check the interfacial interactions between PIM-1 and AlFFIVE-1-Ni, a key parameter to ensure the processability of continuous and mechanically stable membranes and to further shed light on the interface pore nanostructuring. We revealed that the simulated overall porosity of the composite is made of an ultrasmall MOF core growing continuously toward an irregular larger porosity in the MOF/polymer interface. This suggested that AlFFIVE-1-Ni/PIM-1 might be an optimum MMMOF membrane encompassing an inner highly CO_2_ selective pore and a diffuse porosity at its entrance to enhance the overall CO_2_ transport. Nonequilibrium concentration gradient–driven MD (CGD-MD) simulations on both AlFFIVE-1-Ni/PIM-1 and AlFFIVE-1-Ni/6FDA-DAM composites demonstrated that a diffusive nanostructuring of the MOF/polymer interfacial porosity minimizes the molecular entrance effects toward the ultrasmall channels MOF that decisively leads to an acceleration of the CO_2_ transport not only at the interface but also all along the MOF filler. These computational predictions directed the fabrication of the AlFFIVE-1-Ni/PIM-1 MMMOF membrane and its in-depth characterization unraveled the uniform distribution of orientated-MOF nanosheets in the polymeric matrix. Gas permeability tests confirmed an enhancement of the CO_2_ transport for AlFFIVE-1-Ni/PIM-1 versus AlFFIVE-1-Ni/6FDA-DAM MMMOF membrane compared to their associated polymers. Further gas mixture separation tests revealed that the separation performance of the resultant MMMOF membrane combining high CO_2_ permeability and good CO_2_/CH_4_ selectivity is far above the Robeson upper bound and among the best-performing MMMs reported so far that makes AlFFIVE-1-Ni/PIM-1 MMMOF membrane a very promising candidate for natural gas/biogas purification. In a broader perspective, our findings identify emergent design rules of the MOF/polymer interfacial pore nanostructuring to achieve optimal molecular transport by the next generation of MMMOF membranes while maintaining sufficiently high selectivity.

## RESULTS

### In silico atomistic construction of AlFFIVE-1-Ni/PIM-1 composite

Atomistic model for the AlFFIVE-1-Ni/PIM-1 composite was first built in silico by means of a computational approach integrating quantum and force field molecular simulations we previously developed and validated for a series of MOF/polymer systems (*28*–*30*). A (001) surface MOF slab model that proffers maximum exposure of 1D channels is assembled with a simulated PIM-1 structure to mimic a face-to-face alignment of (001) nanosheets in the polymer matrix as attained experimentally for the uniformly oriented continuous AlFFIVE-1-Ni/6FDA-DAM membrane (see Materials and Methods for the computational details). The resulting MMMOF membrane model illustrated in Fig. 1B comprises the AlFFIVE-1-Ni surface slab model in the middle of the simulation box surrounded by the polymer on both sides. AlFFIVE-1-Ni/PIM-1 exhibits a distinct interfacial nanostructuring compared to AlFFIVE-1-Ni/6FDA-DAM as illustrated in Fig. 1B. The overall interfacial pore network of AlFFIVE-1-Ni/6FDA-DAM was demonstrated in our previous work (*21*) to delimit a relatively regular corridor section of ~4.0 Å width along the MOF surface plane owing to the soft nature of 6FDA-DAM (Fig. 1B, top). Analysis of the radial distribution functions for the different MOF/polymer atom pairs averaged over five different composite configurations (see fig. S4) first demonstrated that 6FDA-DAM does not exhibit any specific interactions with the AlFFIVE-1-Ni surface, all atom pairs being separated by a characteristic distance over 4 Å. This continued MOF/polymer van der Waals interaction controls the adhesion between the two components (Fig. 1C) by ensuring a homogeneous distribution of 6FDA-DAM at the MOF surface with MOF/polymer interface distance distributed from 2.5 to 7 Å as shown in the fig. S5. The scenario differs for PIM-1 with the F-atoms of the MOF surface acting as anchoring sites for this polymer favoring specific interactions with its cyano-groups (see fig. S4 and the associated characteristic distance of ~3 Å). This results into a contorted geometry of the PIM-1 backbone that less well conforms to the MOF surface compared to 6FDA-DAM as illustrated by its broader range of MOF/polymer interface distance that goes till 11 Å (see fig. S5) and a more irregular interfacial pore landscape as shown in Fig. 1B. This lastly leads to a pore distribution/size heterogeneity at the AlFFIVE-1-Ni/PIM-1 interface with the typical presence of localized pores up to 13 Å (see Fig. 1, B and D, and fig. S6) that is in sharp contrast with a smaller interfacial interconnected pore distribution with a predominant size of 4.0 Å (see Fig. 1D) observed for AlFFIVE-1-Ni/6FDA-DAM.

We further analyzed the evolution of the void fraction of the composites in the (*x*,*y*) plane for different *z* values considered along the direction normal to the MOF surface. Figure 1E reports the calculated void fraction for the two composite models illustrated in Fig. 1B within a 20 Å distance away from the reference Ni position in the first layer of AlFFIVE-1-Ni. The profile calculated for AlFFIVE-1-Ni/6FDA-DAM emphasizes that the void is concentrated in a narrow range of *z* distance from 3 to 5 Å and sharply decreases above, signifying a sudden loss of porosity in the MOF/polymer region when the distance from the MOF surface exceeds 5 Å. The (*x*,*y*) plane porosity mapping drawn for different *z* values within an interval of 0.5 Å (see Fig. 1F and fig. S7) confirms these findings in line with the interfacial porosity that mostly spreads in a corridor section as illustrated in Fig. 1B. Conversely, for AlFFIVE-1-Ni/PIM-1, the porosity expands in a much broader range of *z* distances up to about 10 Å and then gradually decreases in the *z* = 10 to 20 Å region (see Fig. 1E and fig. S9). This trend is illustrated by the dependence of the porosity mapping provided in Fig. 1F. In this scenario, the pore structuring of AlFFIVE-1-Ni/PIM-1 composite can be seen as an ultrasmall MOF core growing continuously toward a larger porosity in the MOF/polymer interface before decreasing once approaching the polymer region as illustrated in Fig. 1B. This simulated interfacial pore nanostructuring for AlFFIVE-1-Ni/PIM-1 is expected to favor a more effective molecular diffusion pathway from the polymer to the MOF channel entrance, as demonstrated below. This overall distinct pore structuring of AlFFIVE-1-Ni/PIM-1 and AlFFIVE-1-Ni/6 FDA-DAM is observed over the five independent MOF/polymer configurations for the two composites examined in figs. S8 and S10, respectively.

### CO_2_ distribution and transport in the AlFFIVE-1-Ni/polymer composites

**Fig. 2.**
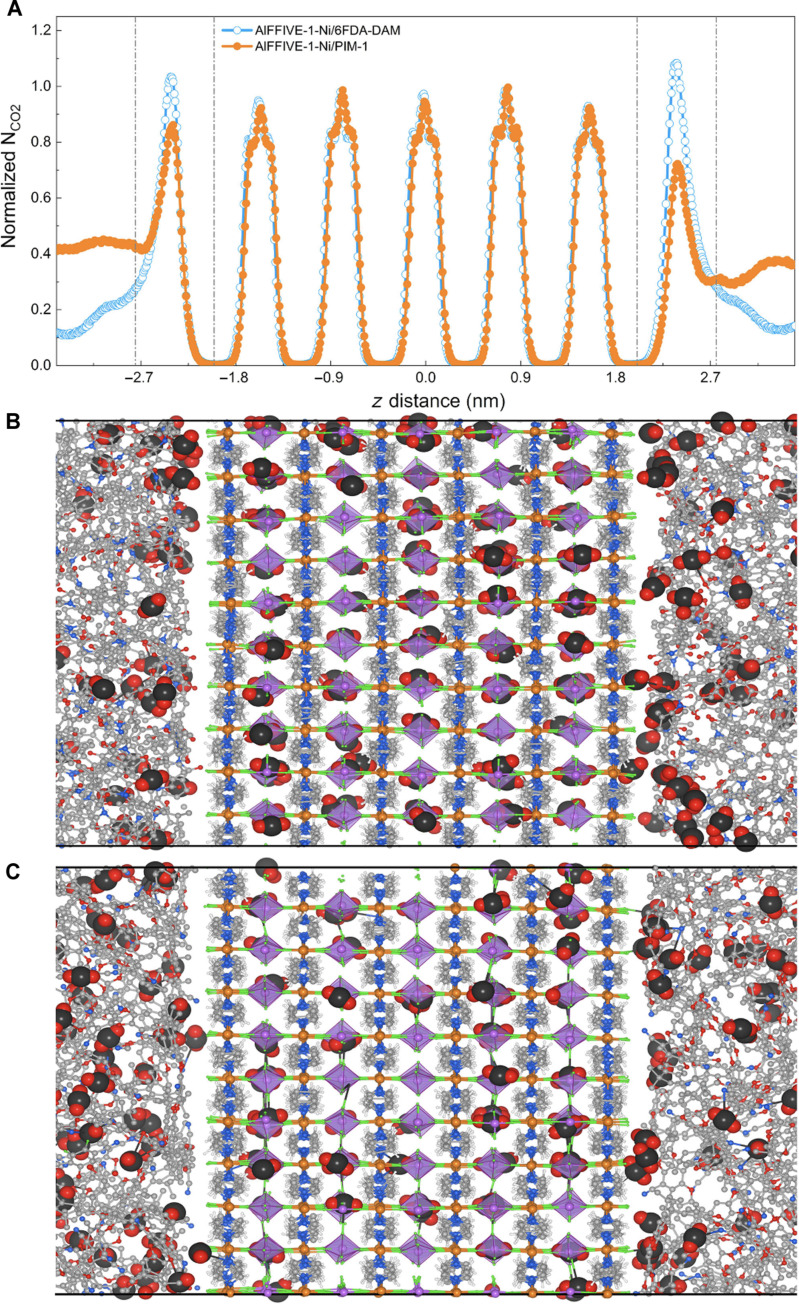
Analysis of CO_2_ distribution in the MOF/polymer composites by E-MD simulations. (**A**) E-MD simulated distribution of the CO_2_ molecules along the *z* direction (*z* denotes the direction along the MOF channel axis and *z* = 0 corresponds to the center of ALFFIVE-1-Ni) for the AlFFIVE-1-Ni/6FDA-DAM and ALFFIVE-1-Ni/PIM-1 composite models. The number of CO_2_ molecules is normalized with respect to the number of molecules present in the AlFFIVE-1-Ni region. The MOF/polymer junction region represented here was defined by the limit between the *z* value for the atomic density of polymer starts to oscillate around an equilibrium value and the *z* value of the center position of first metal (Ni)–pyrazine square-grid layer of ALFFIVE-1-Ni in the MMMOF membrane as defined in our previous studies (junction length of 8.5 Å for both composites-see the atomic density profiles plotted in figs. S13 and S14) (*21*, *32*, *34*)*.* (**B** and **C**) are illustrative snapshots for the CO_2_-loaded systems in both composites. To evidence more clearly the CO_2_ profile at the MOF/polymer junction, the polymers far from the interface were not represented in the figure. Their total polymer lengths along *z* direction are 40.0 ± 1.8 Å and 57.5 ± 2.4 Å for AlFFIVE-1-Ni/6FDA-DAM and ALFFIVE-1-Ni/PIM-1 composite models, respectively.

Equilibrium MD simulations were further conducted to explore the CO_2_ distribution profile along the two atomistic composite models reported in Fig. 1. These composites were first loaded by Monte Carlo simulations at 1 bar CO_2_ pressure (see the Supplementary Materials for computational details). The interfacial region for both composites is accessible to CO_2_ thus ensuring a junction path between the polymer and the MOF channel (Fig. 2A). Notably, a higher concentration of CO_2_ molecules accumulates in the interface of AlFFIVE-1-Ni/6FDA-DAM compared to AlFFIVE-1-Ni/PIM-1 associated with a partial occupancy of the corridor section along the MOF surface as illustrated in Fig. 2B. This high interfacial CO_2_ density and the resultant strong CO_2_/CO_2_ interactions (as shown in figs. S11 and S12) are expected to hinder the reorientation of the molecules toward a required alignment along the channel of AlFFIVE-1-Ni to enable an effective passage throughout the ultrasmall pore MOF entrance.

**Fig. 3.**
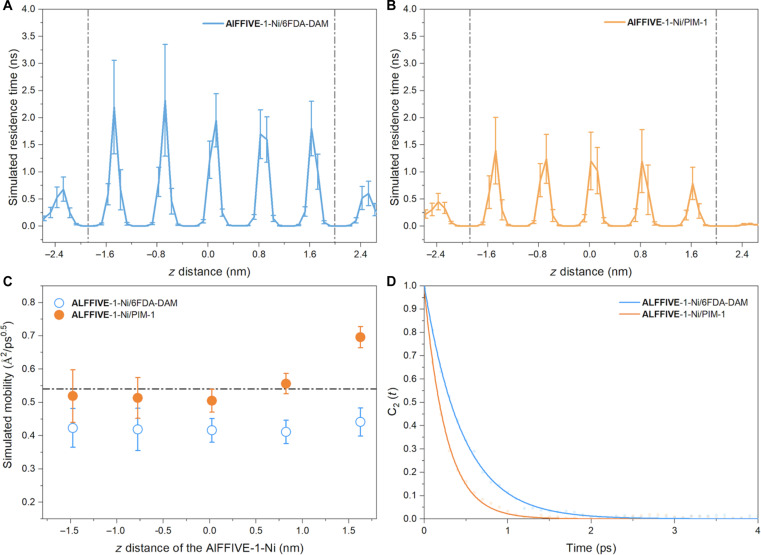
CO_2_ transport in the MOF/polymer composites by CGD-MD simulations. CGD-MD–simulated CO_2_ residence time distributions along the different regions of the composites AlFFIVE-1-Ni/6FDA-DAM (**A**) and AlFFIVE-1-Ni/PIM-1 (**B**). MOF/polymer junctions are shown between dashed lines. The MOF/polymer junction region represented here was defined by the limit between the *z* value for the atomic density of polymer starts to oscillate around its equilibrium value and the *z* value of the center position of first metal (Ni)–pyrazine square-grid layer of AlFFIVE-1-Ni in the composite. Note that the *z* distance values are shifted compared to Fig. 2 since in the CG-MD simulations 10-nm void spaces are added to both sides of the membrane along the *z* direction. (**C**) Simulated *z* dependence of the single file mobility factor for CO_2_ in AlFFIVE-1-Ni for both AlFFIVE-1-Ni/6FDA-DAM and AlFFIVE-1-Ni/PIM-1 composites using the time lag approximation applied to single-file diffusion scenario. The dashed line reports the *F* value of 0.55 Å^2^/ps^0.5^ obtained for AlFFIVE-1-Ni from E-MD simulations (see the MSD plot in fig. S22). (**D**) Orientational autocorrelation function for CO_2_ averaged over all molecules present in the MOF/polymer junction for both composites.

In the case of AlFFIVE-1-Ni/PIM-1, the more diffuse MOF/polymer interfacial porosity enables a much more balanced distribution of CO_2_ between the polymer and the junction (Fig. 2, A to C). This scenario suggests an effective gas flow control throughout the polymer and the junction zones to prevent a detrimental large accumulation of CO_2_ at the interface and thus favoring a drastic reduction of the MOF entrance dissipation for optimum gas transport. CGD-MD simulations were further performed to shed light on the CO_2_ transport along the two composites. These calculations are based on larger and unwrapped composite models as illustrated in fig. S15 and the creation of a concentration gradient between the inlet (feed) and the outlet (permeate) sides of the composites to mimic experimental nonequilibrium conditions applied to membranes (*31*). The inlet and outlet pressures were arbitrarily fixed to 10 bar and to vacuum, respectively, to ensure an effective sampling throughout the consideration of CGD-MD runs for a total of 2.5 ms (i.e., 500-ns MD runs for five different configurations; see figs. S15 to S17 for details). Figure 3A first reveals that the simulated residence time for CO_2_ at the AlFFIVE-1-Ni/PIM-1 junction (around 400 ps) is substantially decreased by ~50% as compared to AlFFIVE-1-Ni/6FDA-DAM junction (around 600 ps). This translates into a substantial enhancement of the calculated interfacial CO_2_ diffusivity along the *z* direction by about the same factor (from 0.9 to 1.3 10^−8^ cm^2^·s^−1^) (table S4) evaluated from the lag time approximation (*32*) (*l*^2^/*t* where *l* is the distance covered by the molecule over the time *t* spent in the MOF/polymer junction) and averaged over all CO_2_ molecules effectively present in the MOF/polymer junction over the MD time. This prediction confirms that a more diffuse MOF/polymer interfacial pore nanostructuring contributes to accelerate the CO_2_ transport at the AlFFIVE-1-Ni/PIM-1 junction toward the MOF channel entrance. This interfacial geometry enables to drastically limit the lateral motions of CO_2_ along the MOF surface as evidenced by the interfacial mean squared displacement (MSD) plotted for CO_2_ in the lateral and *z* directions for AlFFIVE-1-Ni/PIM-1 (see figs. S18 and S19). This is in sharp contrast with the calculated CO_2_ dynamics simulated in the corridor-like AlFFIVE-1-Ni/6FDA-DAM interface where the lateral motions are substantial (see figs. S20 and S21) at the origin of a longer simulated residence time for CO_2_ at the interface. Therefore, the shape of the AlFFIVE-1-Ni/PIM-1 interfacial porosity directs CO_2_ toward an optimum pathway along the direction of the MOF channel to reduce the so-called entrance effects. Notably, Fig. 3 (A and B) reveals that the simulated residence time for CO_2_ in the MOF channel is also considerably reduced in AlFFIVE-1-Ni/PIM-1 versus AlFFIVE-1-Ni/6FDA-DAM, highlighting that the shape of the MOF/polymer interfacial porosity equally modulates the molecular transport throughout the MOF filler. In-depth exploration of the CO_2_ dynamics in AlFFIVE-1-Ni incorporated into both composites unravels a single-file diffusion mechanism that excludes mutual passages of the molecules along the MOF ultrasmall pore channel, i.e., the molecules are confined to advancing one behind the other in the same *z* direction. Here, the MSD for CO_2_ increases with *t*^1/2^ contrary to the normal 1D diffusion where MSD increases with *t*. Figure S22 reports the simulated single-file mobility factor (*F*) profile for CO_2_ along the pristine AlFFIVE-1-Ni MOF calculated from the fitting of the MSD plot [MSD(*t*) = 2*Ft*^1/2^] using E-MD simulations. The *z*-dependent single-file mobility factor in the MOF section of the two composites was further evaluated from the CO_2_ residence time distributions (Fig. 3, A and B) and the associated length covered by CO_2_ using the lag time approximation we proposed to adapt to a single-file diffusion regime (see tables S6 and S7). *F* was proved to be enhanced by ~30% in the first layer of AlFFIVE-1-Ni for the PIM-1–based MOF composite (0.56 Å^2^/ps^0.5^) versus its 6FDA-DAM derivative (0.42 Å^2^/ps^0.5^). This observation emphasizes that a fine-tuning of a pore shape nano structuring of the MOF/polymer interface enables to not only accelerate the dynamics in the MOF/polymer junction but equally along the MOF channel. Figure 3C also evidences that the CO_2_ dynamics becomes gradually faster along the MOF channel in the AlFFIVE-1-Ni/PIM-1 composite owing to a cooperative propagation of the initial acceleration at the MOF entrance that is made possible by the highly correlated single-file diffusion mechanism. This subsequently induces a faster CO_2_ diffusivity at the AlFFIVE-1-Ni/PIM-1 junction located at the outlet compared to AlFFIVE-1-Ni/6FDA-DAM (see table S4). The orientational autocorrelation function (*33*) [C_2_(*t*)] for CO_2_ averaged over all molecules present in the MOF/polymer junction is plotted in Fig. 3D for the two composites. These calculations revealed that the reorientational time scale for CO_2_ is notably faster in the junction of AlFFIVE-1-Ni/PIM-1 (260 fs) as compared to that of AlFFIVE-1-Ni/6FDA-DAM (450 fs) (see table S5). Therefore, the shape of the AlFFIVE-1-Ni/PIM-1 interfacial porosity favors a more effective molecular reorientation, to a priori direct CO_2_ toward an optimum pathway along the direction of the MOF channel to reduce the so-called entrance effects. Therefore, this whole set of simulations demonstrates that a more diffuse interfacial MOF/polymer pore nanostructuring is expected to boost the overall CO_2_ transport in the constructed MMMOF membrane. One should bear in mind that no direct quantitative correlation can be made between neither the simulated CO_2_ interfacial residence times nor the simulated CO_2_ interfacial diffusivity/CO_2_ single-file mobility factor in the MOF pore with the expected CO_2_ gas permeability measured experimentally for the corresponding MMOF membranes since these atomistic composite models do not describe the complexity of the overall MMMs. However, these computational findings enabled us to qualitatively anticipate that the corresponding AlFFIVE-1-Ni/PIM-1 MMMOF membrane is expected to present attractive CO_2_ permeability performance.

### Fabrication of [001]-oriented AlFFIVE-1-Ni/PIM-1 MMMOF membrane and gas separation

**Fig. 4.**
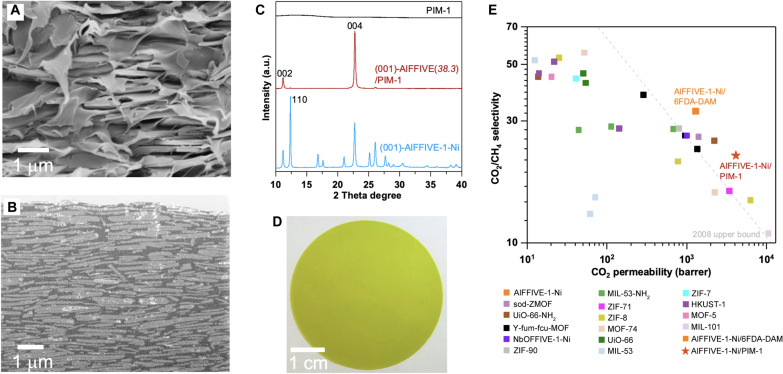
Characterization and gas separation properties. (**A**) Cross-section SEM image and (**B**) focused ion beam SEM image of [001]-oriented AlFFIVE-1-Ni/PIM-1 membrane with 38.3 wt % (001) nanosheets loading. (**C**) XRD patterns of (001)-AlFFIVE1-Ni nanosheets powder, [001]-oriented AlFFIVE-1-Ni/PIM-1 membrane, and PIM-1 polymer. (**D**) Photograph of the corresponding membrane. (**E**) Plot of CO_2_/CH_4_ selectivity versus CO_2_ permeability from a recent literature review of polymer/MOF-based MMMOF membranes (table S8).

This computational prediction inspired us to fabricate [001]-oriented AlFFIVE-1-Ni/PIM-1 MMMOF membrane and perform subsequent gas separation testing. The in-plane alignment of (001) AlFFIVE-1-Ni nanosheets in the PIM-1 polymer matrix was achieved by using our previously reported procedure “slow evaporation-induced in-plane alignment of nanosheets” (*21*). The cross-section SEM image of [001]-oriented AlFFIVE-1-Ni/PIM-1 membrane is shown in Fig. 4A. While the membrane is deliberately broken into pieces for SEM imaging purposes, the polymer fell apart from adjacent nanosheets that generate trenches. However, the SEM image reveals that the nanosheets are uniformly and perpendicularly oriented throughout the membrane along the gas diffusion direction. AlFFIVE-1-Ni/PIM-1 MMMOF membrane shows remarkably similar SEM image (Fig. 4A) as compared to AlFFIVE-1-Ni/6FDA-DAM-DAT and AlFFIVE-1-Ni/6FDA-DAM MMMOF membranes (*21*). This interesting membrane microstructure could be originated from the innate nature of oriented MOF nanosheet mixed-matrix membranes. The highly aligned nature of nanosheets on an extensive area was further characterized using focused ion beam SEM (FIB-SEM) analysis. The FIB-SEM image evidence that the majority of nanosheets are uniformly and in-plane aligned throughout the membrane (Fig. 4B). These analyses corroborate a homogeneous dispersion of a large concentration of MOF nanosheets (38.3 wt %) in the polymeric matrix and an excellent nanosheets-polymer interface compatibility. X-ray diffraction (XRD) pattern of associated membranes exhibit only two major Bragg diffractions, which can be indexed as the (002) and (004) crystallographic planes of AlFFIVE-1-Ni structure, none of the additional diffractions of the free nanosheets crystallites counterpart are detected, corroborating the proper in-plane alignment of (001)-nanosheets inside the polymer matrix and the successful fabrication of uniform [001]-oriented macroscopic AlFFIVE-1-Ni/PIM-1 continuous membrane (Fig. 4C). A photograph of AlFFIVE-1-Ni/PIM-1 MMOF membrane is shown (Fig. 4D).

We conducted single and mixed gas CO_2_ and CH_4_ permeation on AlFFIVE-1-Ni/PIM-1 and pure PIM-1 polymeric membranes (fig. S23 and table S8). The permeation data and the relative permeability enhancement of associated MMMOF membranes when MOF nanosheets were embedded in the two polymer matrices are summarized in table S10. (001) AlFFIVE-1-Ni/6FDA-DAM MMMOF membrane showed a CO_2_ permeability improvement of 414 barrer as compared to the pure 6FDA-DAM membrane. Notably, (001) AlFFIVE-1-Ni/PIM-1 MMMOF membrane presents a substantial CO_2_ permeability enhancement of 2342 barrer as compared to pure PIM-1 membrane. This relative permeability enhancement versus pure polymer membrane is higher for AlFFIVE-1-Ni/PIM-1 versus AlFFIVE-1-Ni/6 FDA-DAM (61.7% versus 46.9%), which supports the predicted acceleration of CO_2_ transport to be assigned to the interfacial pore nanostructuring of the MMMOF membranes. Increasing (001) MOF nanosheets content into the PIM-1 matrix prompted a remarkable increase in CO_2_ permeability and an improvement in CO_2_/CH_4_ selectivity (fig. S23 and table S9). AlFFIVE-1-Ni(38.3 wt %)/PIM-1 demonstrate concurrently 54% permeability and 14% selectivity enhancement under mixed gas (CO_2_/CH_4_:50/50) separation as compared to PIM-1. The relative CO_2_/CH_4_ selectivity enhancement in this report is not as high as in the AlFFIVE-1-Ni/6FDA-DAM MMMOF membrane (*21*) to gain more insight into this behavior, we reviewed the recent literature to better understand the CO_2_/CH_4_ separation performances of MOFs/PIM-1 MMMOF membranes (fig. S24 and table S10). UiO-66/PIM-1 MMMOF membrane demonstrated CO_2_ permeability improvement by substantially lessening the CO_2_/CH_4_ selectivity (*34*). This is a typical example of a MMMOF membrane with a nonideal MOF/polymer interface nanostructuring as evidenced earlier (*35*). In this system, the MOF/polymer compatibility was improved by considering the functionalized UiO-66-NH_2_ since the ─NH_2_ groups are prone to strength the interactions with the PIM-1 polymer (*36*). In this case, CO_2_/CH_4_ selectivity was increased however at the expense of a drop of CO_2_ permeability. On the other hand, ZIF-8/PIM-1 (*37*) and MIL-101/PIM-1 (*38*) MMMOF membranes demonstrated CO_2_ permeability enhancements, while no selectivity improvements were observed. Figure S24 summarizes that the concurrent enhancement of CO_2_ permeability and CO_2_/CH_4_ selectivity and in a MMMOF membrane using PIM-1 as a polymer matrix is remarkably challenging because PIM-1 has remarkably high CO_2_ permeability (3796 barrer); therefore, the gas molecule can easily pass through the polymer phase rather than the molecular sieve MOF fillers. Yet, the separation performance of AlFFIVE-1-Ni/PIM-1 MMMs is still remarkable considering the peculiarity of its components, i.e., highly selective AlFFIVE-1-Ni and highly permeable PIM-1. This positions our MMMOF membrane design among the top-performing MMMOF membranes for CO_2_ capture, far above the Robeson upper bound as shown in Fig. 4E and table S11.

## DISCUSSION

In summary, the diffuse interfacial pore nanostructuring of AlFFIVE-1-Ni/PIM-1 composites was computationally predicted to favor a more effective molecular passage from the polymer region toward the entrance of the selective MOF filler. Nonequilibrium MD revealed that this specific interfacial pore geometry enables an optimum guided path for the molecules along the direction of the MOF channel to reduce the so-called entrance effects, leading to an overall acceleration of the molecular transport in the corresponding MMMOF membrane. This computational prediction directed the fabrication of the [001]-oriented nanosheets AlFFIVE-1-Ni/PIM-1 MMMOF membrane with an ideal in-plane alignment and extremely high loading of [001] MOF nanosheets in the polymeric matrix. The resultant MMMOF membrane was proved to exhibit exceptional CO_2_ permeability while maintaining a relatively good CO_2_/CH_4_ selectivity that makes this membrane among the top-performing MMMOF membranes for CO_2_ capture. This proof of concept highlights the great potential of tailoring MOF/polymer interface in MMMOF membranes that goes beyond the current paradigm of controlling solely the fabrication of homogeneous membranes. These emergent design rules of the MOF/polymer interfacial pore nanostructuring pave the way toward the manufacturing of next-generation MMMOF membranes with maximized permeability and selectivity performance to address a range of gas mixture separations.

## MATERIALS AND METHODS

### In silico construction of the atomistic composite models

The two AlFFIVE-1-Ni/PIM-1 and AlFFIVE-1-Ni/6FDA-DAM composite models were built by applying a methodology implying density functional theory calculations and force field–based MD simulations. The geometry of the AlFFIVE-1-Ni structure was optimized using the Perdew-Burke-Ernzerhof (PBE) functional within the generalized gradient approximation (*39*) in the VASP code (*40*). Details of the two models can be found in the Supplementary Materials. The resulting models of dimensions 45.8 × 45.8 × 110 Å^3^ for AlFFIVE-1-Ni/PIM-1 system and 45.8 × 45.8 × 85 Å^3^ for AlFFIVE-1-Ni/6FDA-DAM were subsequently equilibrated by a series of equilibrium MD simulations in the NVT and the N*P_z_*T ensembles, where *P_z_* corresponds to the pressure component in the *z* direction, i.e., the direction perpendicular to the MOF surface slab. Both polymer and MOF models were considered as fully flexible with the parameters described in the Supplementary Materials. These MD simulations were performed using the LAMMPS package (*41*, *42*). In the force field–based calculations, the AlFFIVE-1-Ni slab model was treated as a fully flexible framework with intramolecular potential parameters taken from the universal force field (UFF) (*43*). The 12-6 Lennard-Jones (LJ) parameters were taken from the generic force fields DREIDING (*44*) and UFF for the atoms of the organic and inorganic nodes, respectively. The polymer model was treated as fully flexible: bonded interactions were modeled by using the analytical expression and parameters described by the generic force field GAFF (*45*), while the nonbonded interactions were treated by 12-6 LJ potential contributions and coulombic terms. The guest CO_2_ molecule was described by the EPM2 model, corresponding to 3 atom–centered charged LJ sites (*46*).

#### E-MD simulations

The two CO_2_-loaded AlFFIVE-1-Ni/PIM-1 and AlFFIVE-1-Ni/6FDA-DAM composite models initially generated by Grand Canonical Monte Carlo simulations were considered for further E-MD simulations in the NVT ensemble performed by using GROMACS-2019.4 package (*47*).

### CGD-MD simulations

Gas transport through the composite models was modelled by CGD-MD simulations using the GROMACS-2019.4 package (*47*) patched with a modified PLUMED-2 (*48*) enhanced sampling plug-in. The temperature was set to 1200 K [corresponds to β^−1^ = 2.38 kcal/mole in energy scale versus 40 kcal/mole in earlier works (*49*)] in all CGD-MD simulations to accelerate the dynamics, which is a common practice to accelerate infrequent and rare events (*49*, *50*). The conclusions gained at high temperature were validated by the experimental design of the MMMOF membrane with improved CO_2_ permeability compared to its 6FDA-DAM derivative. Schematic representation of the CGD-MD simulation setup and associated parameters are provided in fig. S15 and table S3. In this simulation, a concentration gradient is created between the feed and the permeate sides of the composite model to capture the nonequilibrium conditions in the typical experimental setup and steady gas transport versus simulation time is maintained (see figs. S16 and S17). In all simulations, the composite models were placed in the center of the simulation box and 10-nm void spaces were added to both sides of the membranes along *z* direction. Atoms within 0.5 nm from both ends of the composite model were tethered to their initial *z* coordinates to prevent their drifting due to the guest concentration gradient. Periodic boundary condition was applied in all directions. Simulations were run in the NVT ensemble and the temperature of the systems was controlled using a Nosé-Hoover thermostat (*51*). The thermostat coupling constant was set to 0.1 ps. Separate thermostats were used for fluid molecules and individual parts of the composite to thermalize every component of system. Particle mesh Ewald method (*52*) was used to account for long-range electrostatic interactions. A 12-Å cutoff distance was used for the van der Waals interactions and the real part of the Ewald summation. Five independent configurations were considered for the two composites to achieve a better sampling. The equations of motion were integrated with a 1 fs time step using a Verlet scheme during 500 ns for each configuration. Residence time distributions for CO_2_ were calculated over every five considered configurations and averaged. Total simulation time reached to 2.5 ms for these CGD-MD simulations.

### Void fraction analysis

This analysis was performed through the sampling of the empty space present in the two atomistic composite models within a 20 Å distance away from the MOF surface along the *z* axis. For each *z* value, the 50 × 50 Å *xy* plane is scanned. A grid spacing of 0.5 Å was considered in all three directions and the free porosity region was defined as the nodes in the grid spacing devoid of any atoms. Only those grid nodes situated at a distance larger than the sum of the atomic radius and an additional 0.5 Å were considered in the calculation of the void fraction.

#### Synthesis of (001) AlFFIVE-1-Ni nanosheets

Nanosheets of AlFFIVE-1-Ni were synthesized using a method described in a prior study (*21*). Pyrazine (C_4_H_4_N_2_, 1.80 g, 22.03 mmol) was dissolved in a 2:1 (v/v) ethanol-water mixture (9 ml). Nickel acetate tetrahydrate [Ni(OCOCH_3_)_2_·4H_2_O, 2.20 g, 8.84 mmol] was then added to the solution, followed by sonication for 2 min.

In a separate container, aluminum hydroxide [Al(OH)_3_, 0.32 g, 4.10 mmol] was dissolved in a 3:1 (v/v) HF-water mixture (4 ml). These solutions were combined and sonicated for 3 min, resulting in a sky-blue mixture. The solvothermal reaction was carried out at 50°C for 3 days under static conditions. The resulting precipitate was collected via centrifugation at 5000 rpm, washed with a total of 300 ml of water, and subsequently rinsed with 60 ml of ethanol. Last, the nanosheets were dispersed in the required amount of CHCl_3_ solution.

#### (001) AlFFIVE-1-Ni nanosheets stock solution

To prevent agglomeration, the nanosheets were not dried before membrane casting. The concentration of nanosheets in the CHCl_3_ solution was determined by drying a 1-ml aliquot to measure the mass of nanosheets. The resulting stock solutions were found to have a concentration of 96 mg/ml.

#### Synthesis of PIM-1 polymer

PIM-1 was synthesized from a condensation reaction between 5,5′,6,6′-tetrahydroxy-3,3,3′,3′-tetramethyl-1,1′-spirobisindane (6.8 g, Sigma-Aldrich) and tetrafluoroterephthalonitrile (4 g, TCI) in the presence of K_2_CO_3_ (6 g, Sigma-Aldrich) and anhydrous dimethylformamide (150 ml, Sigma-Aldrich). The solution was continuously stirred under nitrogen atmosphere at 65°C for 72 hours. The resulting solution was then cooled and poured into deionized water (500 ml). The solid product was subsequently dissolved in chloroform and reprecipitated from methanol, filtered, and dried under vacuum at 120°C overnight.

#### Membrane fabrication

The process of membrane fabrication involved working within a glove bag containing CHCl_3_ vapor to hinder the quick evaporation of the solvent from the developing membrane. Before conducting gas adsorption and permeation assessments, the membranes were subjected to thermal treatment at 200°C for 20 hours under dynamic vacuum conditions to ensure the removal of any remaining solvent from both the polymer and MOF pores.

#### Pure PIM-1 membrane

PIM-1 powder was first dried in a dynamic vacuum oven for 12 hours at 150°C. A pure PIM-1 polymer membrane with the anticipated thickness (typically 70 μm) was produced by dissolving 200 mg of dried PIM-1 in CHCl_3_ (3.5 ml). The solution was agitated using a mechanical shaker for 4 hours to ensure complete dissolution of the polymer powder. Subsequently, the solution was decanted into a glass petri dish on a leveled surface, and the dish was then placed in a glove bag presaturated with CHCl_3_ vapor for at least 30 min. The film was left in the glove bag 12 hours to facilitate the slow evaporation of the CHCl_3_ solvent.

#### Fabrication of [001]-oriented AlFFIVE-1-Ni/PIM-1 membrane

A membrane composed of AlFFIVE-1-Ni/PIM-1 oriented along the (001) plane was synthesized via a technique termed slow evaporation-induced in-plane alignment. This method entailed dispersing (001) AlFFIVE-1-Ni nanosheets within a polymer matrix of PIM-1. Membranes with varying loadings of (001) nanosheets were created by dissolving specified amounts of PIM-1 and (001) AlFFIVE-1-Ni nanosheets in CHCl_3_ solvent, resulting in membrane thicknesses ranging from 50 to 70 μm.

For the preparation of the PIM-1 polymer solution, 150 mg of dried PIM-1 was dissolved in 2 ml of CHCl_3_. The dissolution process involved shaking on a mechanical shaker for 3 hours at room temperature.

The suspension of MOF nanosheets was prepared by dispensing the required volume of aliquot from the stock solution of (001) AlFFIVE-1-Ni nanosheets (1.25 ml for a 38.3 wt % membrane) into a 25-ml glass vial. Then, 0.75 ml of CHCl_3_ was added to maintain a total volume of 2 ml.

The MOF suspension was mixed with 10% of the polymer solution and stirred for 30 min (referred to as priming). Subsequently, the remaining polymer solution was slowly introduced into the MOF suspension and stirred overnight at 35°C. The resulting casting solution was poured into a glass dish placed on a leveled surface within a glove bag saturated with CHCl_3_ vapor for at least 30 min. The dish was left within the glove bag overnight at room temperature to allow for the gradual evaporation of the CHCl_3_ solvent. Last, the freestanding [001]-oriented AlFFIVE-1-Ni/PIM-1 membrane was dried in a vacuum oven at 200°C for 20 hours to eliminate any residual CHCl_3_.

#### Nanosheets and [001]-oriented AlFFIVE-1-Ni/PIM-1 membrane characterizations

##### SEM imaging of nanosheets and membrane

The structure of AlFFIVE-1-Ni nanosheets and their arrangement within MMMOF membranes were examined using scanning electron microscopy (SEM) analysis conducted with a field-emission SEM, specifically the FEI Nova Nano SEM450. Operating parameters included an acceleration voltage of 3 kV and an emission current of 40 pA. Sample preparation for SEM involved dispersing the nanosheets in ethanol and depositing them onto aluminum SEM pin stubs via drop-casting. To capture cross-sectional SEM images, MMMOF membranes were fractured cryogenically in liquid nitrogen to preserve their microstructures. To prevent charge buildup, the sample underwent sputter-coating with approximately 3 nm of iridium.

#### FIB-SEM analysis of [001]-oriented AlFFIVE-1-Ni/PIM-1 membrane

FIB-SEM experiments were performed using Helios G4 UX DualBeam microscopes from FEI. The FIB was operated at 5 kV and 25 pA, milling slices with a nominal thickness of 50 nm. A total of 100 to 235 individual SEM micrographs were captured from consecutive cross sections exposed during milling. These micrographs were acquired at magnifications ranging from 12,000 to 45,000, using a secondary electron detector operating at 1 kV. Subsequently, the stack of images underwent alignment to an external feature present on the membrane surface, facilitated by a cross-correlation algorithm. Furthermore, a stretching operation in the *y* direction was conducted to correct for foreshortening resulting from the tilt angle between the specimen cross section and the SEM detector.

#### Determination of MOF structure and MOF nanosheets orientation in membrane

The nanosheets’ structure and orientation were determined through XRD analysis. XRD data were obtained using a Bruker D8 Advance diffractometer (Cu Kα λ = 1.54056 Å) in Bragg-Brentano θ-θ geometry. The instrument was operated at 40 kV/40 mA with an automatic divergence slit (irradiated length = 0.6 mm) and used a flat plate sample holder. Data collection was performed at room temperature using the continuous-count method (3° min^−1^) within the range of 2θ = 4° to 40°.

#### Gas permeation measurements

Single and mixed gas permeations were conducted using our previously reported procedure (*21*).

## Supplementary Materials

### This PDF file includes:

Supplementary Text Figs. S1 to S24 Tables S1 to S11 Legends for data S1 and S2 References
