# Supplementary material for: Tuning MOF/polymer interfacial pore geometry in mixed matrix membrane for upgrading CO2 separation performance
Source: Sci Adv. 2024 Jul 10;10(28):eadk5846. doi: 10.1126/sciadv.adk5846 (PMC11235163; doi:10.1126/sciadv.adk5846)
Supplement: Supplementary file 1 — Supplementary Text Figs. S1 to S24 Tables S1 to S11 Legends for data S1 and S2 References [file sciadv.adk5846_sm.pdf]

Science Advances
AAAS

Supplementary Materials for

Tuning MOF/polymer interfacial pore geometry in mixed matrix membrane for upgrading CO_2 separation performance

Aydin Ozcan *et al.*

Corresponding author: Guillaume Maurin, guillaume.maurin1@umontpellier.fr;
Mohamed Eddaoudi, mohamed.eddaoudi@kaust.edu.sa

Sci. Adv. **10**, eadk5846 (2024)
DOI: 10.1126/sciadv.adk5846

The PDF file includes:

Supplementary Text
Figs. S1 to S24
Tables S1 to S11
Legends for data S1 and S2
References

Other Supplementary Material for this manuscript includes the following:

Data S1 and S2

Supplementary Text

The initial step involved the optimization of the crystal structure of **AlFFIVE**-1-Ni by using Density Functional Theory (DFT) calculations, allowing for complete relaxation of both the atomic positions and cell parameters. All these calculations were performed using the Vienna *ab initio* Simulation Package (VASP, version 5.4.4)(40) with a plane wave energy cut-off of 650 eV and convergence criteria of 10^{-5} eV and 10^{-2} eV/Å for the energy and sum of the atomic forces, respectively. The electronic exchange-correlation interaction was treated using the Perdew–Burke–Ernzerhof (PBE) functional within the generalized gradient approximation (GGA).(39) The first Brillouin zone was set with a 3×3×2 Monkhorst–Pack *k*-point mesh. The van der Waals correction DFT-D3 method (53) was also used to accurately account for long-range dispersion interactions. In order to better describe the *d* states of the Ni of **AlFFIVE**-1-Ni, an Hubbard *U* correction of 6.4 eV was used. (54)

Next, the DFT-optimized crystal structure of **AlFFIVE**-1-Ni model was further employed to construct the **AlFFIVE**-1-Ni (001) surface model. The plane (001) was chosen to orient the cleavage of the surface to mimic the experimental scenario corresponding to the fabrication of **AlFFIVE**-1-Ni nanosheets with exposed (001) facets experimentally reported earlier.(21) The slab model has a zero dipole in the direction perpendicular to the surface slab terminated by F-atoms coordinated to the Ni-atoms, as shown in Fig. S1. This surface model was then geometry-optimized at the DFT level using the same level of theory and parameters as for the optimization of the bulk model. The density derived electrostatic and chemical (DDEC06) method, as implemented in the CHARGEMOL module,(55) was employed to calculate the partial atomic charges for the surface model.

A final surface slab model was then constructed for the force field simulations by extending 5 times the surface model in the *x* and *y* directions giving final *x* and *y* lengths of 48.925 Å. In these computations, the surface slab model was regarded as a fully flexible framework employing intra-molecular potential parameters taken from the Universal Force Field (UFF)(43). Intermolecular interactions were modeled using 12-6 Lennard-Jones (LJ) van der Waals and Coulombic contributions. The cutoff is set to 12 Å for all following simulations. The 12-6 LJ parameters were taken from the generic forcefields UFF(47) and DREIDING(44) for the atoms of the inorganic and organic nodes respectively. Fig. S1 provides an illustration of the atom types present in the

AlFFIVE-1-Ni-(001) surface model. (*cf.* ref. 21 for the respective charges and non-bonded parameters).

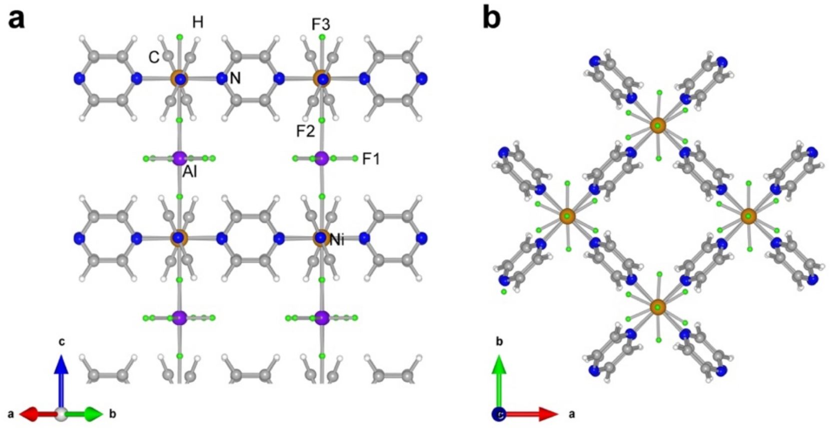

Fig. S1

Atom types associated with the **AlFFIVE**-1-Ni (001) surface model viewed from (a) side and (b) top, respectively. Only a single end of the surface slab structure is shown on the left for clarity. F, N, C, Ni, Al and H are respectively shown in green, blue, gray, orange, purple and white, respectively. This figure highlights that the MOF surface is terminated by F-atoms.

The 6FDA-DAM polymer model utilized in this study was adopted from our previous work.(56) Initially, it was constructed by starting with a united-atom monomer, as depicted in Fig. S2, and undergoing a controlled polymerization process (61). A comprehensive description of this methodology can be found in our earlier publications.(28, 57), The terminations of the resulting polymer were achieved as follows: on one end, the replacement of a nitrogen atom by an oxygen atom and, on the other end, the addition of $–NH_2$ group to a carbon atom (Fig. S3). This polymer model was treated as fully flexible in our calculations: bonded interactions were modeled using the analytical expression and parameters described by the generic forcefield GAFF while the non-bonded interactions were treated by a LJ potential contribution and a coulombic term, with parameters taken from our previous work and reminded in Table S1.

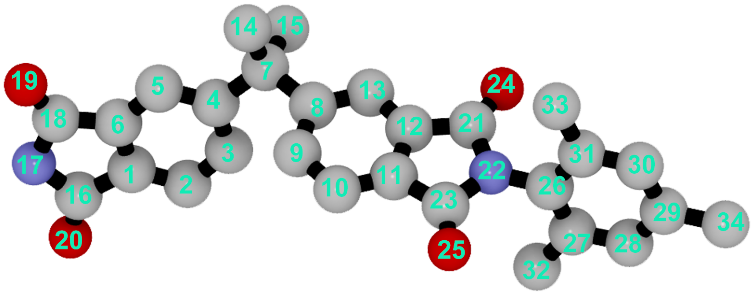

Fig. S2

Optimized 6FDA-DAM monomer structure, with head and tail atoms represented by atoms N17 and C30.

Atom type	Number	ε_{ii} [kcal/mol]	σ_{ii} [Å]	Q_i [e]
CA11	1,11	0.0417	3.88	-0.137
CA12	6,12			-0.023
CA21	2,10			+0.082
CA22	3,9	0.1003	3.695	-0.049
CA23	5,13			-0.039
CA24	28			-0.350
CA3	4,8			+0.305
CA4	26,30	0.0417	3.88	-0.425
CA5	27,29,31			+0.470
CH0	7	0.0010	6.40	-0.502
CF3	14,15	0.0417	4.73	+0.053
CH3	32,33,34	0.1947	3.75	-0.080
COO	16,18,21,23	0.1689	3.72	+0.369
OCO	19,20,24,25	0.3924	3.05	-0.383
NN1	17,22	0.0238	3.78	+0.107
OES	Termination	0.1093	2.80	-0.102
NA1	Termination	0.2206	3.34	-0.660
HA1	Termination	0.0000	0.00	+0.330

Table S1

12-6 LJ parameters and charges for 6FDA-DAM model.

To construct the PIM-1 polymer model, the Polymatic code(57) was also utilized in conjunction with the LAMMPS code(41, 42). The PIM-1 configuration was derived from this initial model by performing annealing cycles via MD simulations in two *NVT* ensembles: NVT_{high} and NVT_{low}. T_{high} and T_{low} were varied between 100 K and 900 K to achieve diverse equilibrium configurations. The detailed process can be found in our previous publications.(28-30, 58) The obtained PIM-1 polymer model consisted of 4 chains of 146 monomers (see Fig. S3 and Table S2).

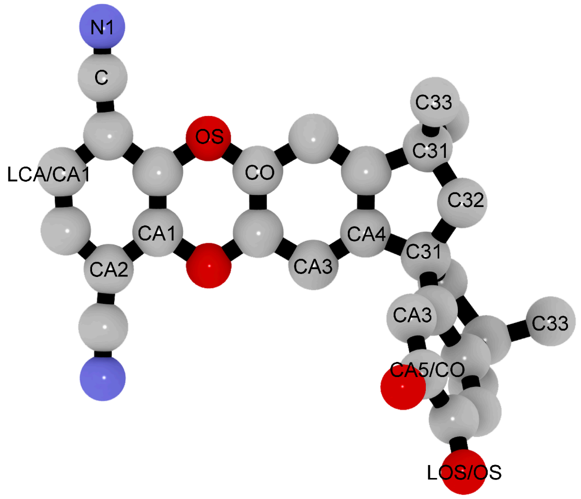

Fig. S3

Scheme of the PIM-1 monomer model.

**Atom types**	ε_{ii} (kcal/mol)	**σii** (Å)	**Q** **(e)**	**Atom types**	ε_{ii} (kcal/mol)	**σii** (Å)	**Q** **(e)**
**CA1**	0.0636	3.600	+0.085	**C31**	0.00104	6.400	+0.562
**CA2**	0.0435	3.880	-0.287	**C32**	0.1059	3.890	-0.280
**CA3**	0.1050	3.695	-0.144	**C33**	0.2033	3.750	-0.133
**CA4**	0.0435	3.880	-0.044	**CO**	0.0636	3.600	+0.285
**CA5**	0.0636	3.600	+0.187	**LCA**	0.0636	3.600	+0.185
**OS**	0.1452	2.600	-0.200	**LOS**	0.2345	3.040	-0.538
**C**	0.1243	3.550	+0.427	**F**	0.2286	2.850	-0.100
**N1**	0.1243	2.950	-0.401	**HOH**	0.000	0.000	+0.436

Table S2.

Atom types, LJ parameters and charges for the PIM-1 model.

The construction of the **AlFFIVE**-1-Ni/6FDA-DAM and **AlFFIVE**-1-Ni/PIM-1 composite models were obtained by using the computational strategy we previously developed and validated on a series of MOF/Polymer systems. First, the atomic coordinates of the polymer model underwent unwrapping in the z-direction, and subsequently, the simulation box was adapted to conform to the lattice parameters of the AlFFIVE-1-Ni (001) structure model, which measured 48.925×48.925×150.000 in dimensions.

Subsequently, the polymer model was brought into contact with the **AlFFIVE**-1-Ni (001) slab model, and subjected to a 21-step molecular dynamics (MD) simulation regimen, which encompassed NVT and NPT cycles. These cycles were controlled using the Berendsen thermostat and barostat, with relaxation times of 0.1 ps and 0.5 ps respectively, within a customized DL_POLY Classic code.(59, 60) This method facilitated the polymer's equilibration, leading to the formation of a compact **AlFFIVE**-1-Ni/polymer composite. The interactions between the MOF surface and the polymer were treated by incorporating van der Waals and Coulombic terms, with the cross 12-6 LJ parameters determined using the Lorentz Berthelot mixing rule.(61) The two components of the composite were treated as fully flexible with the force field parameters mentioned in the previous sections. The van der Waals interactions were handled with a 12 Å cutoff while the electrostatic interactions were calculated using the Ewald summation method with a tolerance of 10^{-6}.(62) These calculations were performed during a total simulation time of 10 nanoseconds (ns) with a time step of 1 femtosecond (fs).

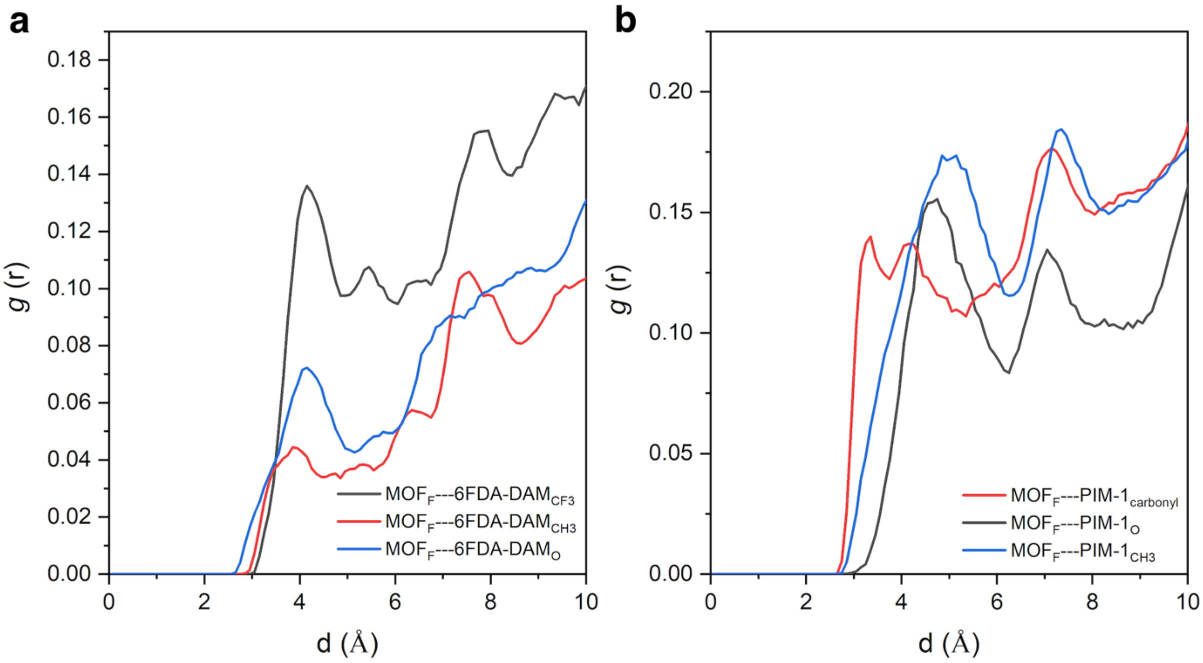

Fig. S4

Calculated radial distribution functions (RDFs) between $F_{AlFFIVE-1-Ni}$ and the atoms of the polymers in the (a) **AlFFIVE-1**-Ni/PIM-1 and (b) **AlFFIVE-1**-Ni/6FDA-DAM composite models, respectively.

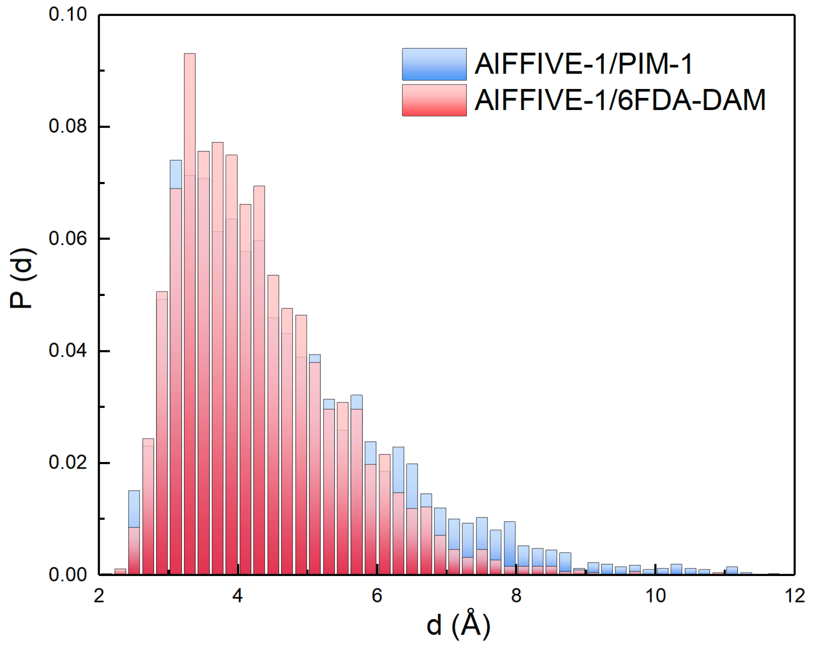

Fig. S5.

Shortest MOF/polymer interface distance distribution for **AlFFIVE-1-**Ni/PIM-1 and **AlFFIVE-1**-Ni/6FDA-DAM composite models averaged over 5 independent configurations.

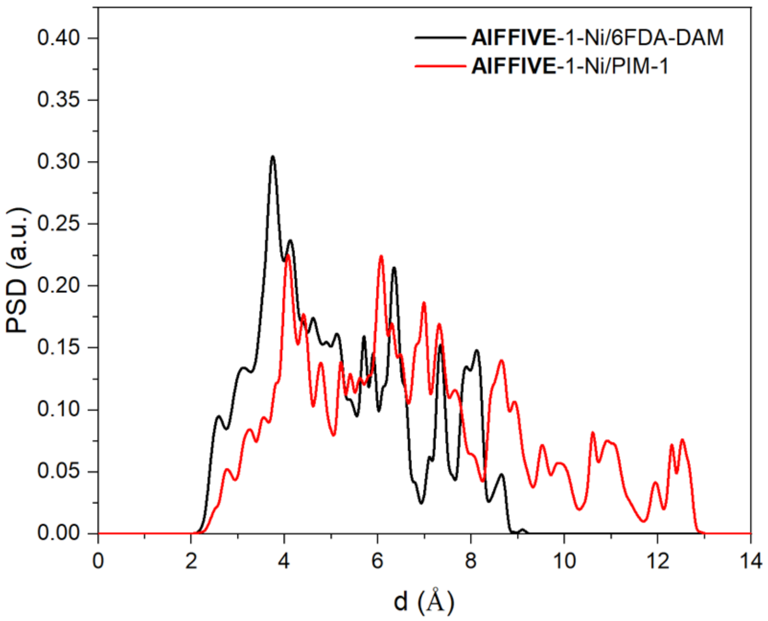

Fig. S6

Calculated pore size distributions (PSD) for the two different composite models. The plot is statistically determined for each composite system five independent MOF/Polymer interface configurations were considered. The PSD calculation is performed for the composite excluding the contribution of the **AlFFIVE**-1-Ni.

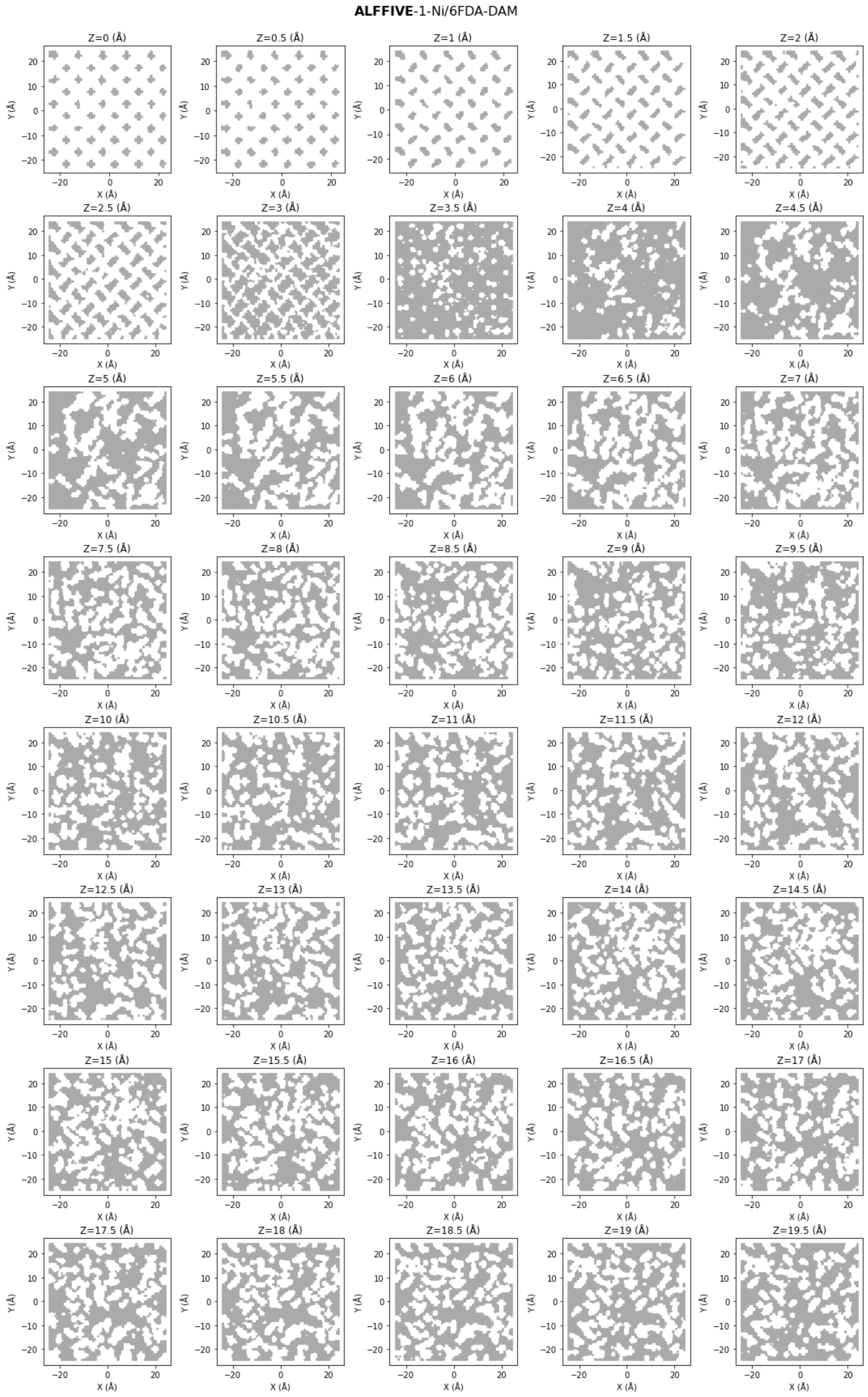

Fig. S7

Illustration of the free porosity over the xy-plane for **AlFFIVE**-1-Ni/6FDA-DAM composite model represented in Fig.1 for different z-values when one moves away from the MOF surface, where gray regions represent void spaces, and white regions denote the presence of polymers.

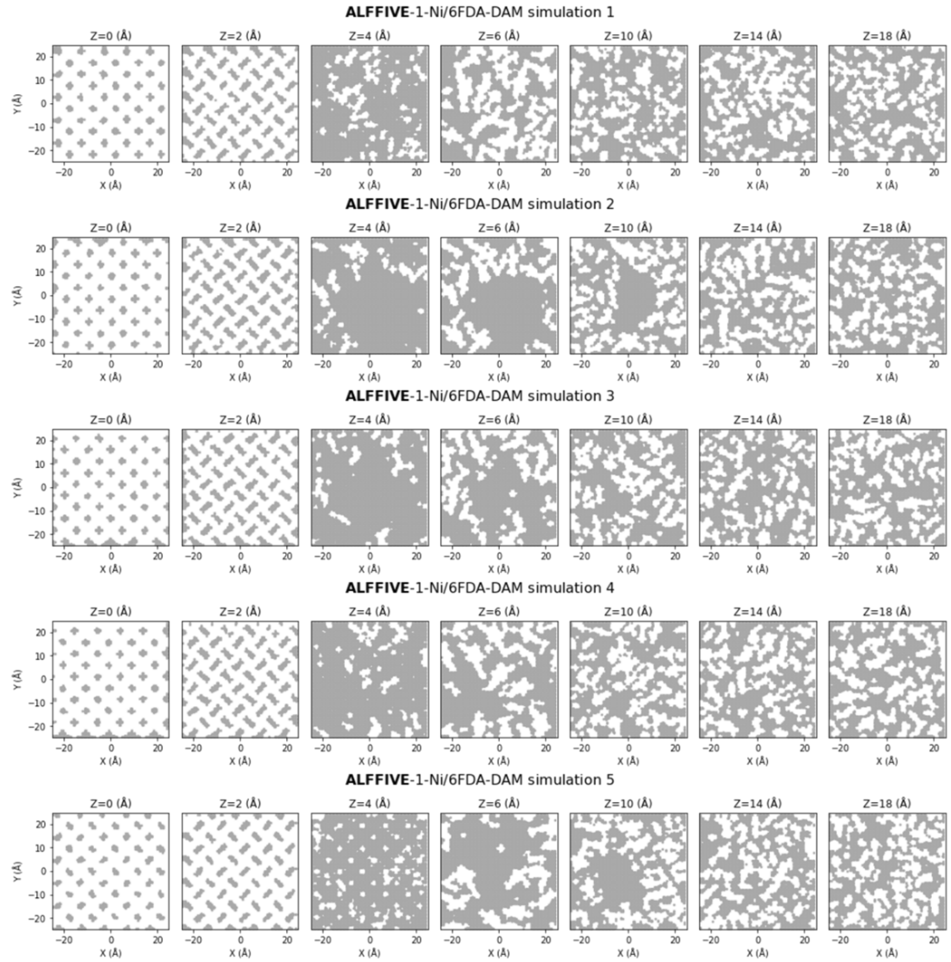

Fig. S8

Illustration of the free porosity over the xy-plane for 5 different configurations of **AlFFIVE**-1-Ni/6FDA-DAM for different z-values when one moves away from the MOF surface, where gray regions represent void spaces, and white regions denote the presence of polymers.

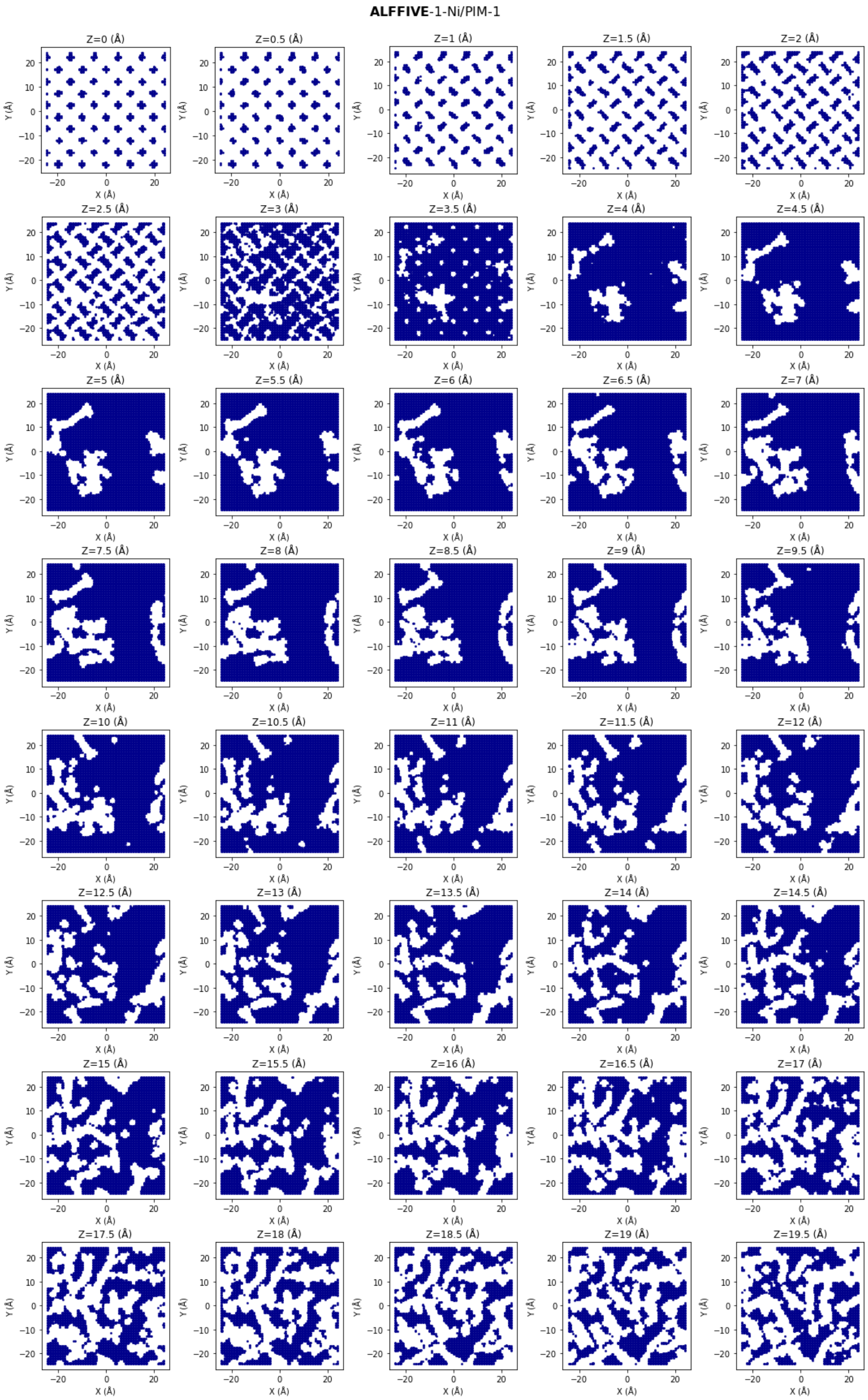

Fig. S9

Illustration of the free porosity over the xy-plane for **AlFFIVE**-1-Ni/PIM-1 composite model represented in Fig.1 for different z-values when one moves away from the MOF surface, where blue regions represent void spaces, and white regions denote the presence of polymers.

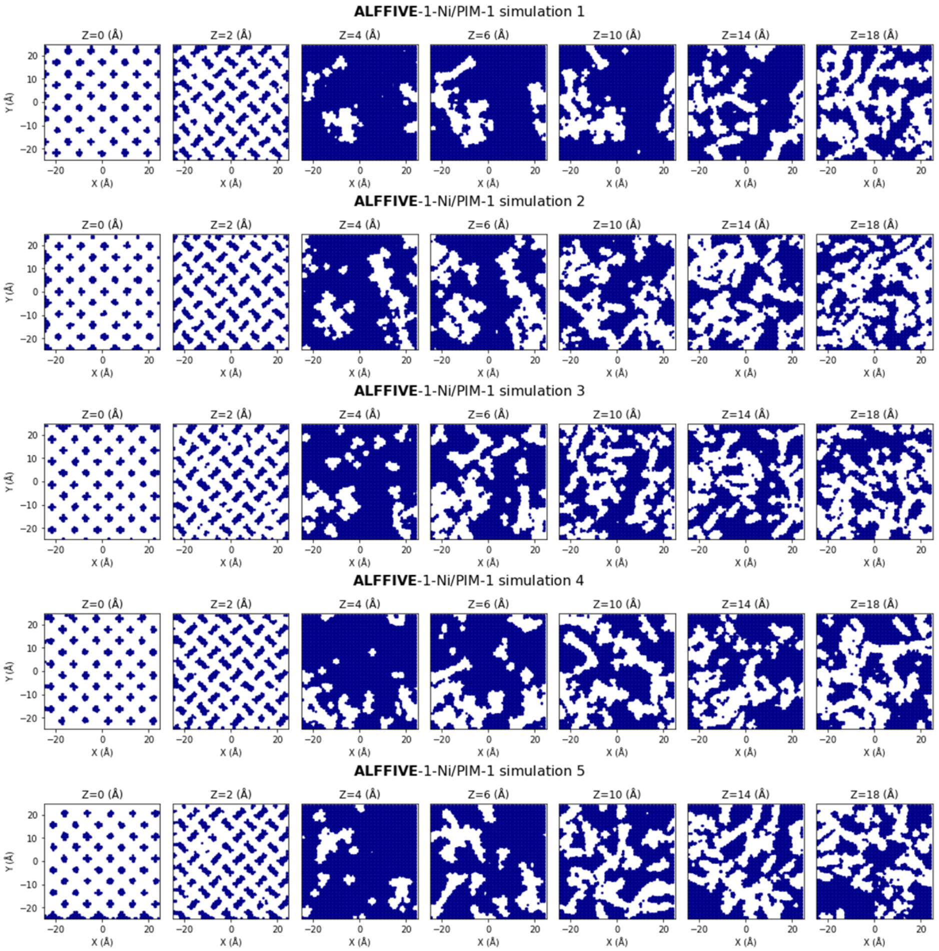

Fig. S10

Illustration of the free porosity over the xy-plane for 5 different configurations of **AlFFIVE**-1-Ni/PIM-1 for different z-values when one moves away from the MOF surface, where gray blue regions represent void spaces, and white regions denote the presence of polymers.

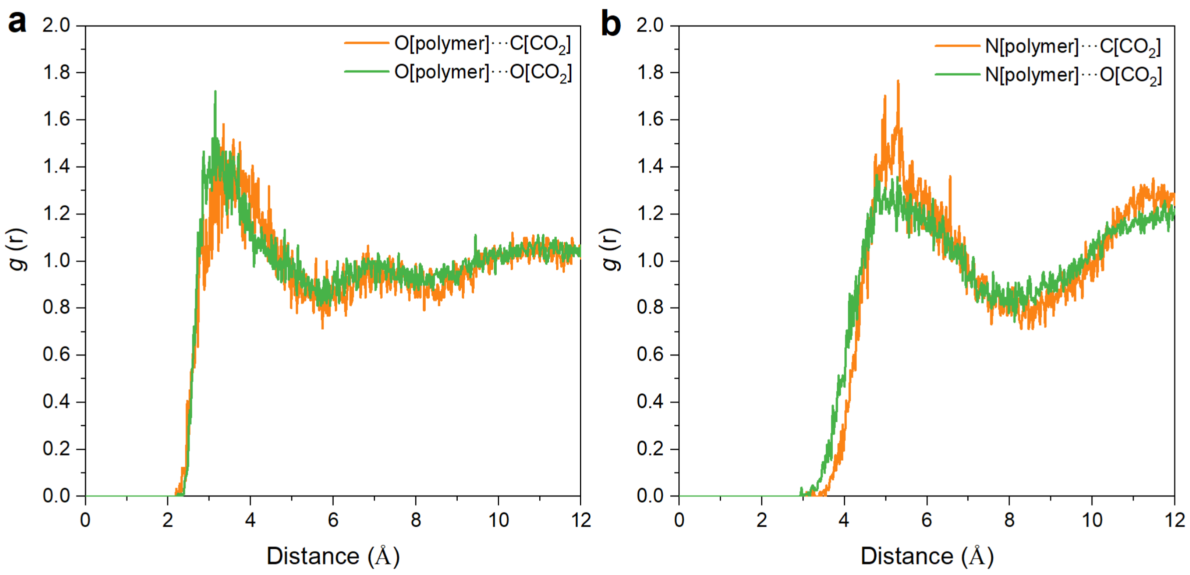

Fig. S11

(**a-b**) Radial distribution function calculated between CO_2 and the Oxygen (carbonyl group, *cf.* Supplementary Fig. S2) and Nitrogen atoms (*cf.* Fig. S2) of 6 FDA-DAM at the interface of the **AlFFIVE**-1-Ni/6FDA-DAM composite. Simulations were carried out at 300K with 1 bar pressure in the corresponding composite.

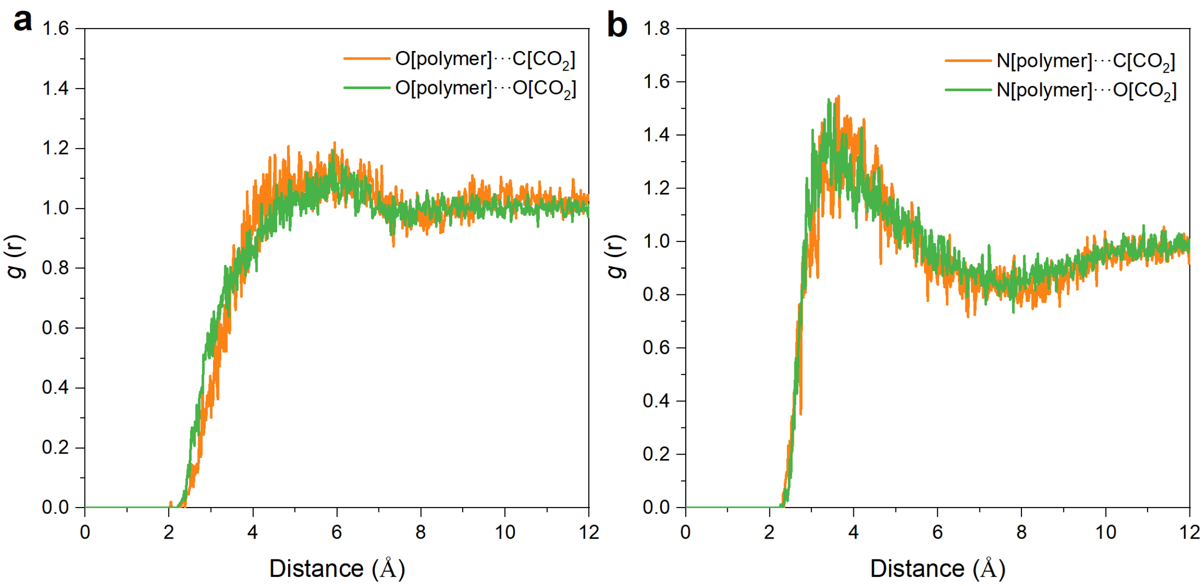

Fig. S12

(**a**-**b**) Radial distribution function calculated between CO_2 and the Oxygen (ether group, *cf.* Supplementary Fig. S3) and Nitrogen (nitrile group, *cf.* Fig. S3) atoms of PIM-1 at the interface of the **AlFFIVE**-1-Ni/PIM-1 composite. Simulations were carried out at 300K with 1 bar pressure in the corresponding composite.

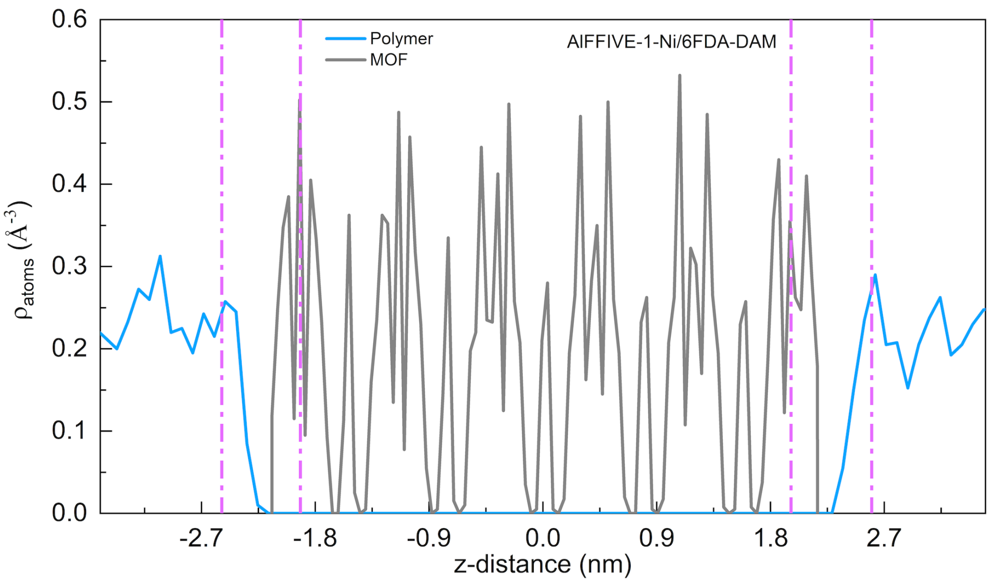

Fig. S13

Atomic density plot of MOF and polymer as a function of z-coordinate for the representative configuration of **AlFFIVE**-1-Ni/6FDA-DAM composite. We remind that the interfacial region represented in dashed lines is defined by the limit between the z value for which the polymer atomic density starts to oscillate around an equilibrium state, and the z value of the center position of first metal(Ni) - pyrazine square-grid layer of **AlFFIVE**-1-Ni in the composite vanishes.

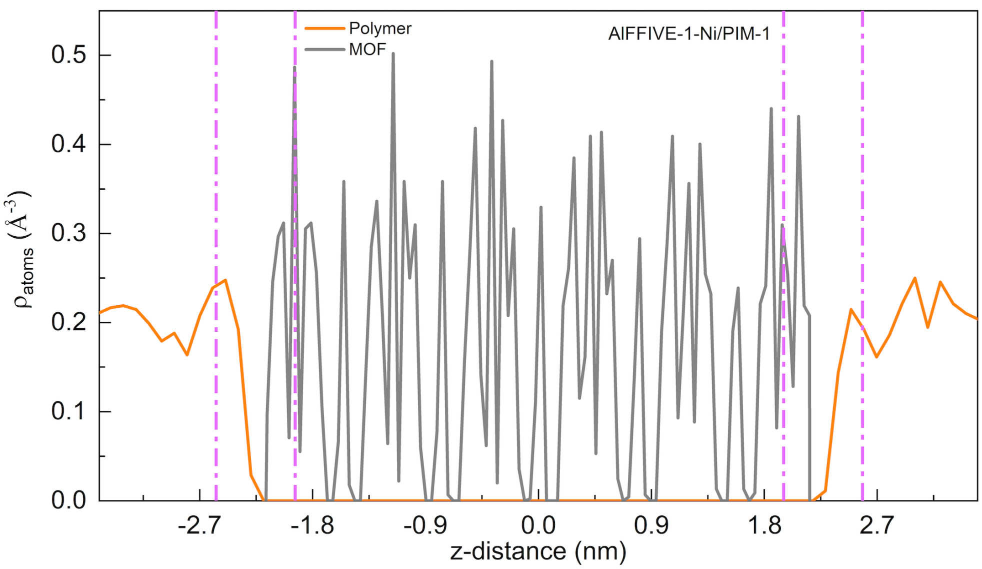

Fig. S14

Atomic density plot of MOF and polymer as a function of z-coordinate for the representative configuration of **AlFFIVE**-1-Ni/PIM-1 composite. We remind that the interfacial region represented in dashed lines is defined by the limit between the z value for which the polymer atomic density starts to oscillate around an equilibrium value and the z value of the center position of first metal(Ni) - pyrazine square-grid layer of **AlFFIVE**-1-Ni in the composite vanishes.

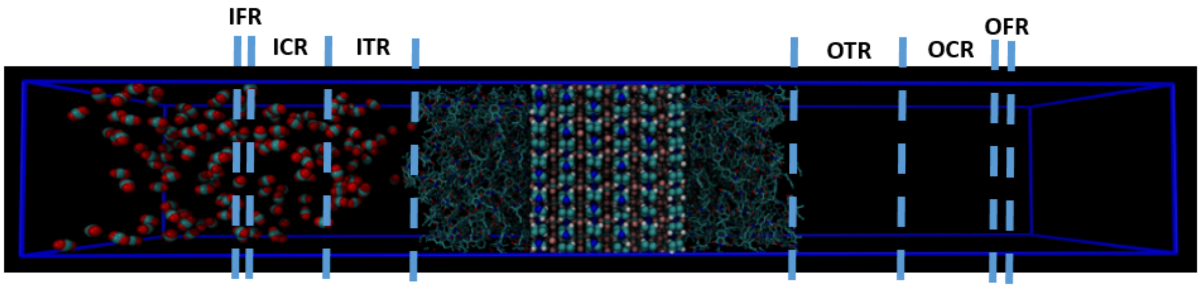

Fig. S15

Schematic representation of the simulation box used in the CGD-MD simulations. IFR=Inlet Force Region, ICR=Inlet Control Region, ITR=Inlet Transition Region; OTR=Outlet Transition Region, OCR=Outlet Control Region and OFR=Outlet Force Region).

The gradient was maintained with adaptive forces located in IFR (inlet force region) and OFR (outlet force region) that enable to control the concentration inside the control volumes (ICR and OCR) to keep a target value. This is achieved by imposing two different target values in the inlet and outlet control region, where the forces are defined by the following equations:

$$F_{inlet} = k_{inlet}(n_{inlet}^{target} - n_{inlet}^{instant})G^{inlet}(z - Z_F^{inlet}, w)$$

$$F_{outlet} = k_{outlet}(n_{outlet}^{target} - n_{outlet}^{instant})G^{outlet}(z - Z_F^{outlet}, w)$$

where F is adaptive bias force for inlet and outlet, respectively, k_{inlet} and k_{outlet} O are force constants, $n_{inlet}^{target}$and are n_{outlet}^{target}target values at ICR and OCR respectively, and $n_{inlet}^{instant}$, $n_{outlet}^{instant}$ are the instantaneous concentrations in the ICR and OCR. G^{inlet} and G^{outlet} are two Gaussian-shaped functions of width w, centered in Z_F^{inlet} and Z_F^{outlet} respectively and G^{inlet} and G^{outlet}serve the purpose of localizing the bias force in the IFR and OFR. In this way, gradient is maintained between both sides of the membrane.

	Side	Z_F (nm)	W (nm)	k_i ($kJnm^3mol^{-1}$)	ICR/OCR width (nm)	ITR/OTR width (nm)
**AlFFIVE-1**-Ni/ 6 FDA-DAM	Inlet	4.875	0.25	10000	2.5	2.5
				100000		
	Outlet	27.275	0.25	10000	2.5	2.5
				100000		
**AlFFIVE-1**-Ni/ PIM-1	Inlet	4.875	0.25	10000	2.5	2.5
				100000		
	Outlet	31.205	0.25	10000	2.5	2.5
				100000		

Table S3

The parameters used in the permeation simulations. Z_F is the centre of external biasing force located region, w is the width of the external biasing force region and k_i is the force constant.

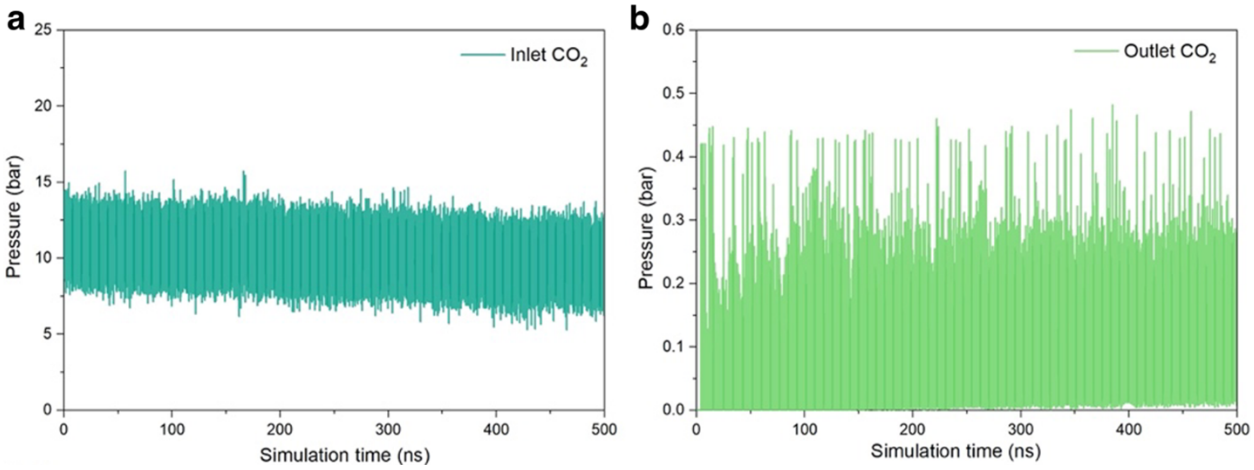

Fig. S16

Averaged pressure control at the (a) inlet and outlet (b) control regions for **AlFFIVE**-1-Ni/6FA-DAM composite model along the 500 ns CGD-MD simulations.

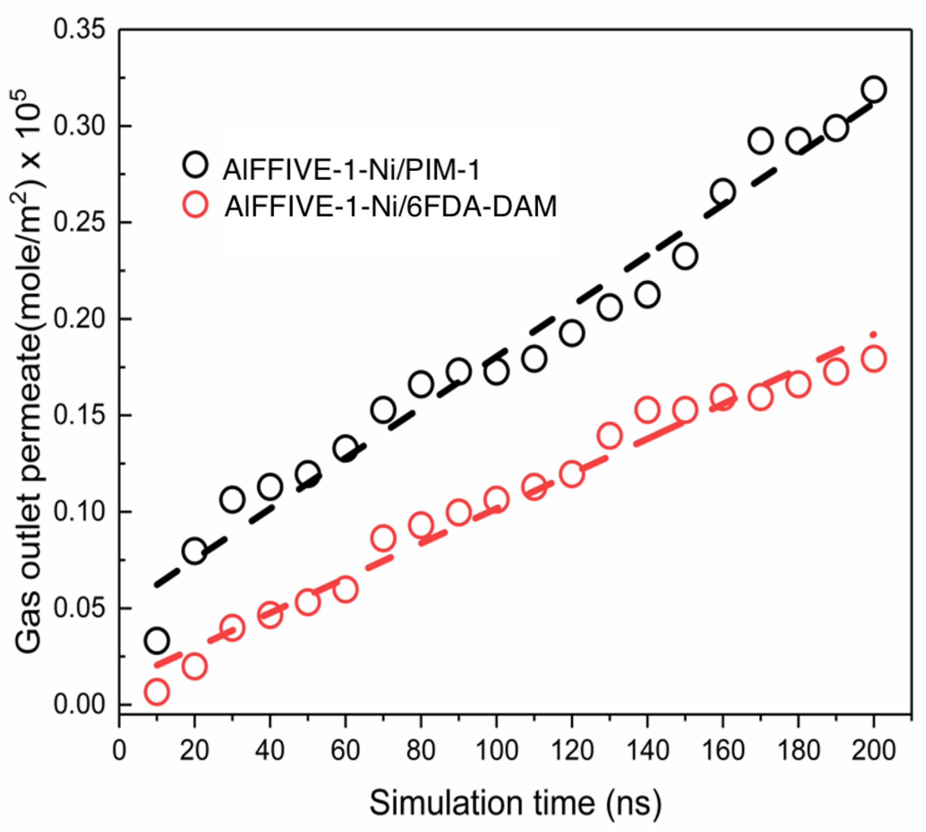

Fig. S17

CO_2 permeation from inlet (feed) to outlet (permeate) over the CGD-MD simulations time for **AlFFIVE-**1-Ni/6FA-DAM and **AlFFIVE-**1-Ni/PIM-1 MMMOF models.

**Diffusivity** ($cm^2.s^{-1}$) × 10^{-8}	**Inlet MOF/polymer heterojunction**	**Outlet MOF/polymer heterojunction**
**AlFFIVE**-1-Ni/PIM-1	1.3 $\pm$ 0.2	11.2 $\pm$ 2.4
**AlFFIVE**-1-Ni/6FDADAM	0.9 $\pm$ 0.15	1.0 $\pm$ 0.19

Table S4

CGD-MD simulated interfacial diffusivity for CO_2 averaged over the inlet and outlet MOF/polymer junction of the **AlFFIVE**-1-Ni/PIM-1 and **AlFFIVE**-1-Ni/6 FDA-DAM composites. The reported values are averaged over the CO_2 molecules spending their times in the inlet and outlet MOF/polymer junctions and over the 5 different configurations considered each for 500 ns MD runs.

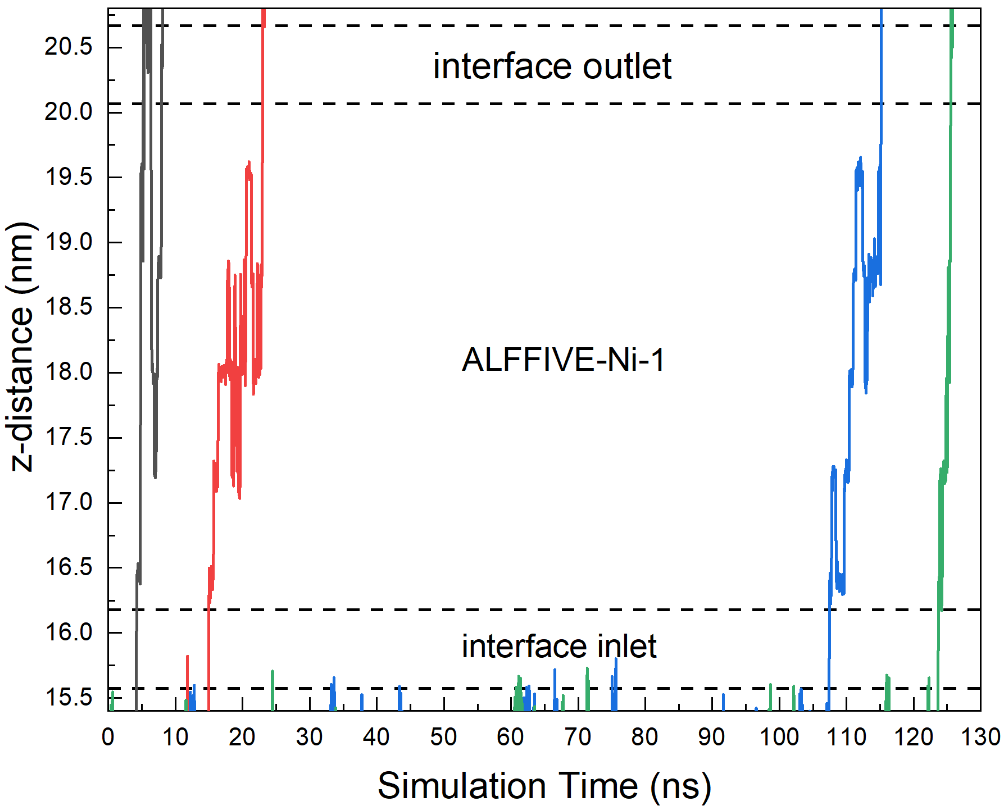

Fig. S18

Representative passage times of molecules through **AlFFIVE**-1-Ni/PIM-1 composite over the CGD-MD simulations.

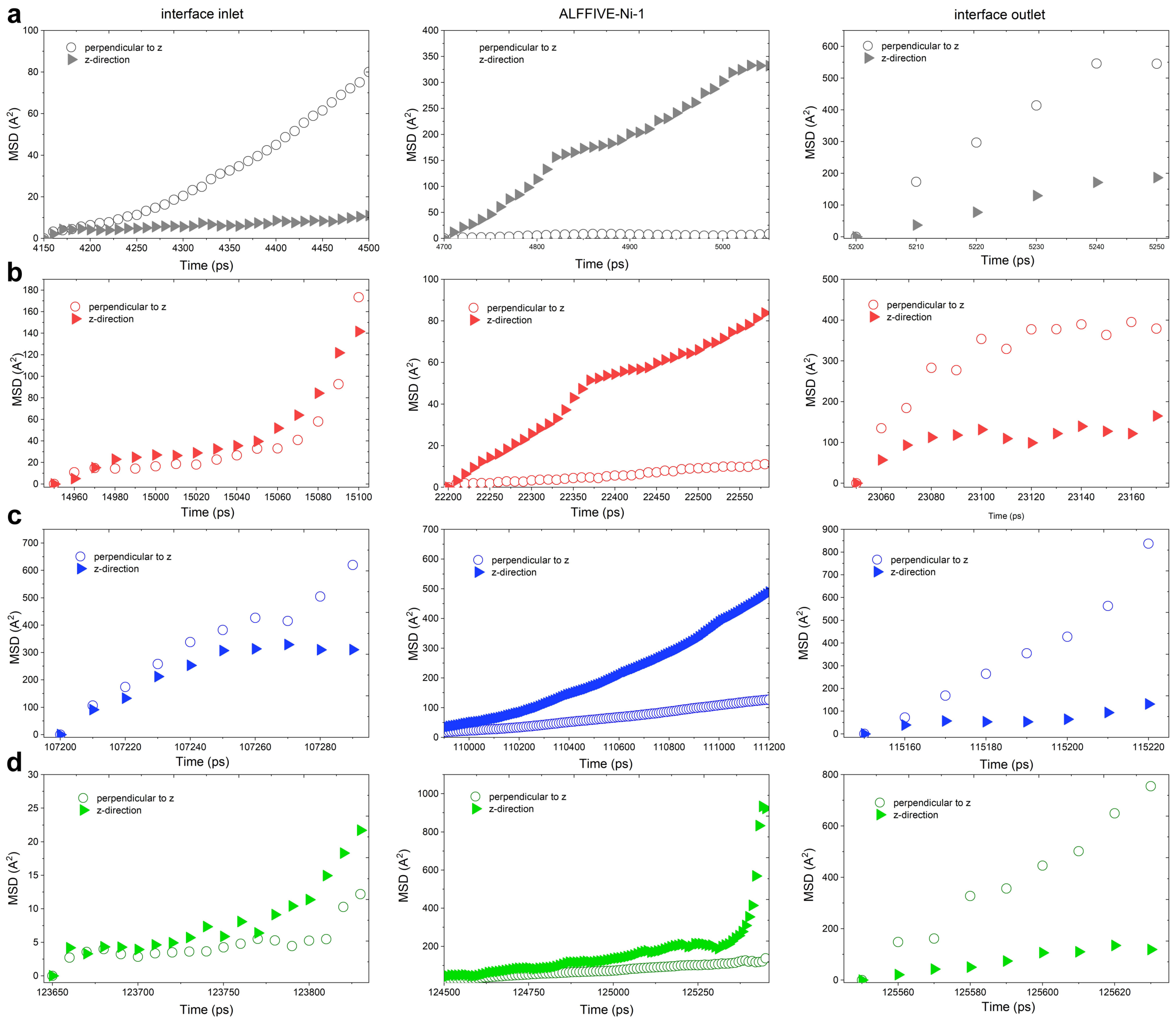

Fig. S19

(a-d) z-direction and lateral MSDs for the selected CO_2 molecules in Fig. S18 through **AlFFIVE-1-Ni/PIM-1** composite over the CGD-MD simulations.

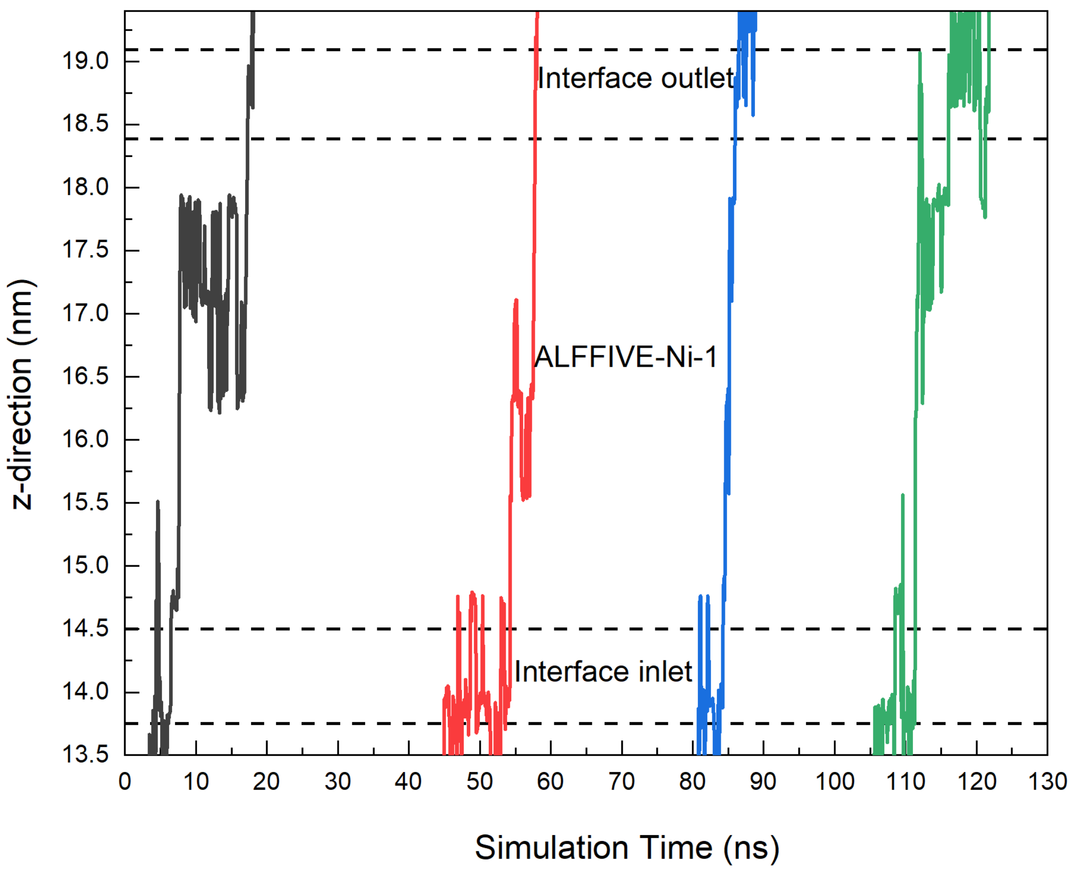

Fig. S20

Representative passage times of molecules through **AlFFIVE**-1-Ni/6FDA-DAM composite - over the CGD-MD simulations.

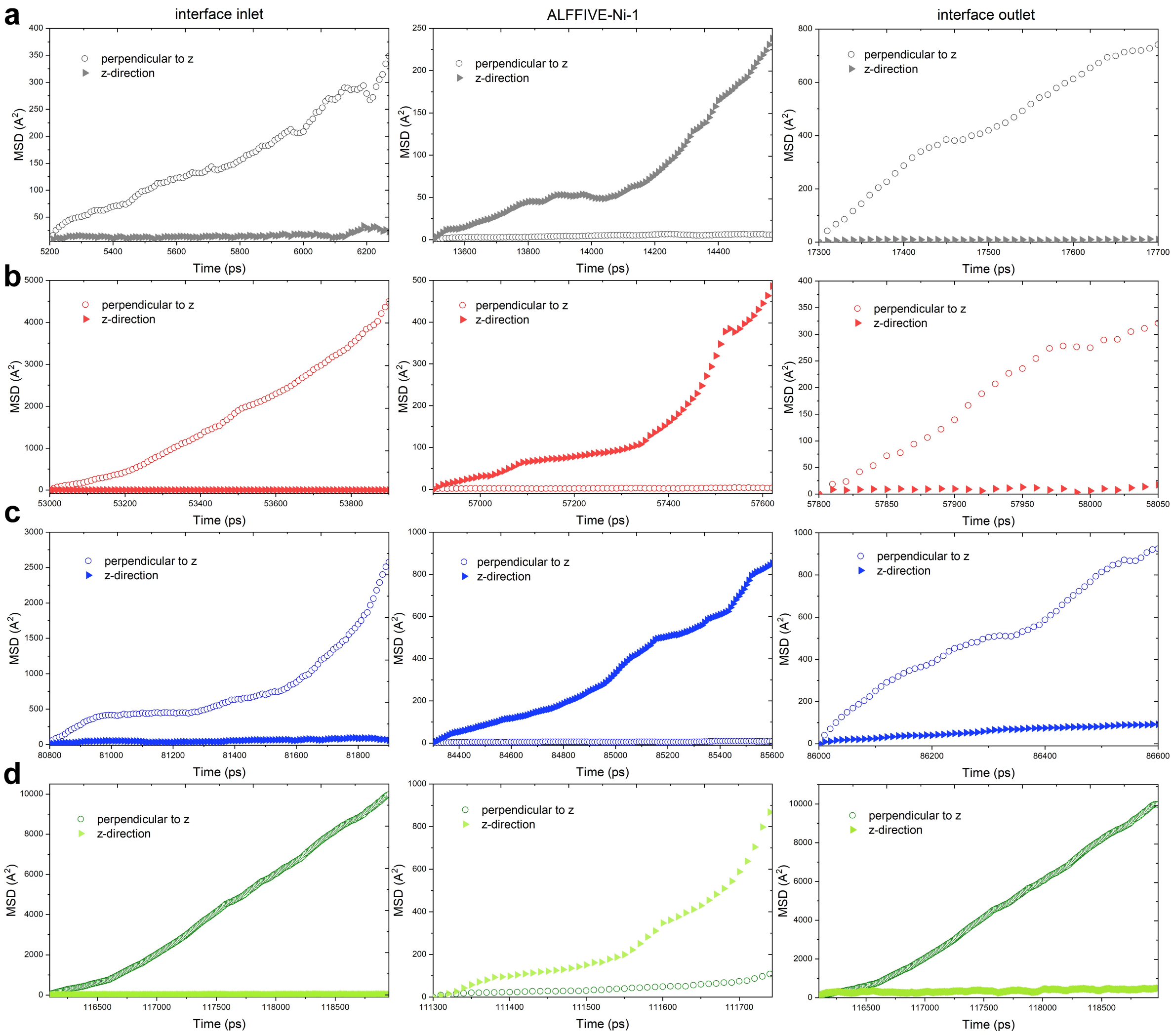

Fig. S21

(a-d) z-direction and lateral MSDs for the selected CO_2 molecules in Fig. S20 through **AlFFIVE-1-Ni/P6 FDA-DAM composite** over the CGD-MD simulations.

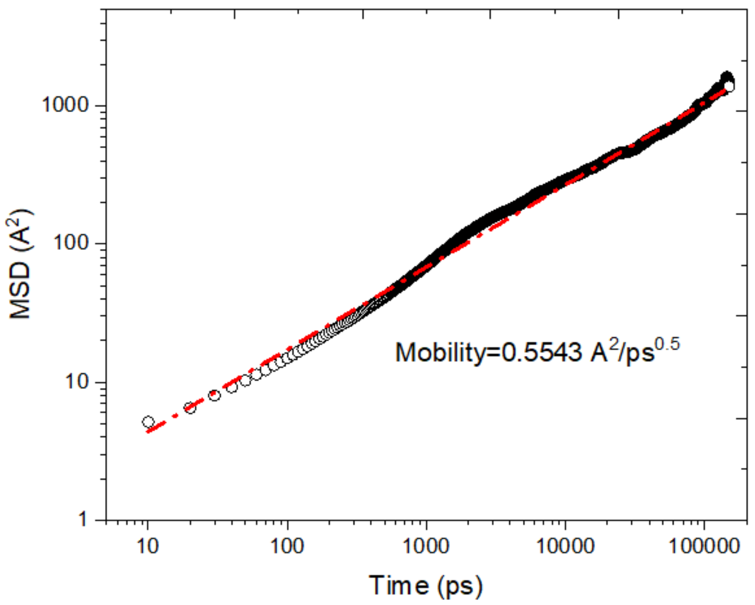

Fig. S22

MSD plot for CO_2 obtained from EMD simulations performed at 1200 K and 10 bar CO_2 pressure for 200 ns (100 ns equilibration followed by 100 ns of production) on the pristine MOF **AlFFIVE**-1-Ni averaged over 5 different MOF configurations.

	A	B	Fitting error (R)
**AlFFIVE**-1-Ni/6FDA-DAM	2.203	1.002	0.007
**AlFFIVE**-1-Ni/PIM-1	3.809	0.988	0.002

Table S5

Exponential fit data for rotational autocorrelation functions of CO_2 at the heterojunction of both composites reported in Figure 3d. The rotational autocorrelation functions are calculated via $C_2(t) = \langle P_2(e(t).e(0))\rangle$ where P_2 is Legendre polynomial of second-rank and e(t) is the unit vector corresponding to orientation of the molecules. The reorientation time scale of CO_2 in both cases can be obtained by fitting $C_2(t)$ by an exponential decay function (Be^{-At}). The corresponding parameters as well as the fitting error R are reported. Relevant time scale for librational motion corresponds to 1/A.

**ALFFIVE**-1-Ni/6FDA-DAM Position(z-direction) (nm)	L_z (nm)	Time (τ) (ns)	Mobility ($A^2/ps^{0.5}$) ($F(z) = \frac{L_z^2}{2\tau^{0.5}}$)
-1.48	0.710	3.54 ± 0.96	0.42 ± 0.05
-0.70	0.725	3.67 ± 1.08	0.418 ± 0.06
0.11	0.720	3.54 ± 0.60	0.415 ± 0.03
0.91	0.715	3.62 ± 0.61	0.411 ±0.03
1.61	0.705	3.17 ± 0.60	0.444 ± 0.04

Table S6

Evaluation of the single-file mobility factor F at difference z- distance of the MOF for **AlFFIVE**-1-Ni embedded in the **ALFFIVE**-1-Ni/6FDA-DAM composite model using a lag-time approximation adapted to single-file diffusion.

**ALFFIVE**-1-Ni/PIM-1 Position(z-direction) (nm)	L_z (nm)	Time (τ) (ns)	Mobility ($A^2/ps^{0.5}$) ($F(z) = \frac{L_z^2}{2\tau^{0.5}}$)
-1.48	0.710	2.39 ± 0.71	0.518 ± 0.07
-0.70	0.725	2.37 ± 0.55	0.513 ±0.06
0.11	0.720	2.50 ± 0.68	0.504 ±0.07
0.91	0.715	2.14 ± 0.74	0.556 ±0.1
1.61	0.705	1.30 ± 0.34	0.695 ± 0.09

Table S7

Evaluation of the single-file mobility factor F at difference z- distance of the MOF for **ALFFIVE**-1-Ni embedded in the **AlFFIVE**-1-Ni/PIM-1 composite model using a lag-time approximation adapted to single-file diffusion

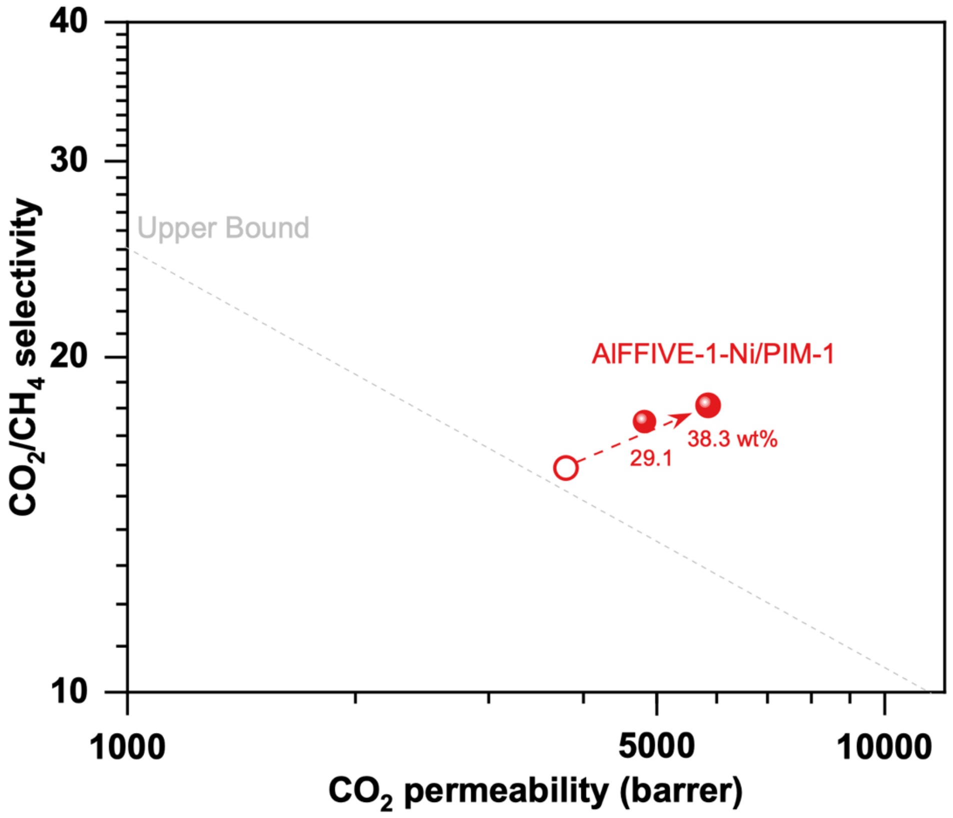

Fig. S23

CO_2 permeability and CO_2/CH_4 selectivity of **AlFFIVE**-1-Ni/PIM-1 MMMOF with various nanosheets loading in wt %. Permeation condition: CO_2/CH_4: 50/50; 2 bar, at 35 °C.

Membrane	CO_2 permeability (barrer)	Permeability enhancement (%)	*Ref.*
6FDA-DAM	881.9	--	(21)
(001)-**AlFFIVE**(*58.9*)/6FDA-DAM	1295.7	46.9	
PIM-1	3796.3	--	*This work*
(001)-**AlFFIVE**(*38.3*)/PIM-1	6138.8	61.7	

Single gas feed pressure: 2 bar; permeation at 35 °C. 1 Barrer = 10^{-10} cm^3 (STP) cm/cm^2 s cmHg.

Table S8

A comparison of the gas separation performances and permeability (P) enhancement of [001]-oriented **AlFFIVE**-1-Ni/6FDA-DAM and **AlFFIVE**-1-Ni/PIM-1 membranes.

Membranes	Single gas permeability (barrer)		CO_2/CH_4 Selectivity	Mixed gas permeability (barrer)		CO_2/CH_4 Selectivity
	CO_2	CH_4		CO_2	CH_4	
PIM-1	3796.3	238.8	15.9	3702.8	245.9	15.1
**AlFFIVE**-1-Ni(*29.1*)/PIM-1	4985.7	294.4	16.9	4818.7	275.5	17.5
**AlFFIVE**-1-Ni(*38.3*)/PIM-1	6098.4	345.6	17.6	5847.5	322.1	18.2

Table S9

Single gas and mixed CO_2/CH_4 separation performances of the **AlFFIVE**-1-Ni/PIM-1 membranes at different nanosheets loading.

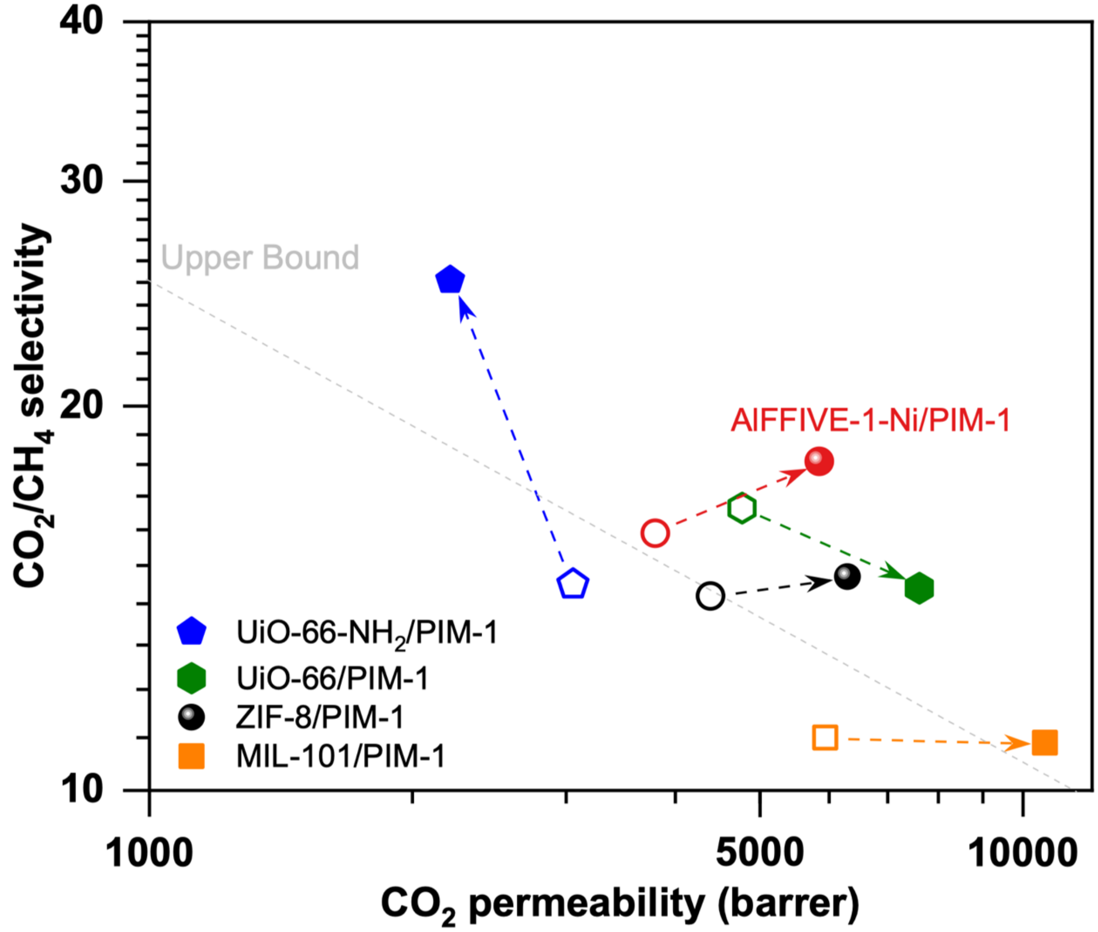

Fig. S24

A plot of the CO_2/CH_4 selectivity *vs* CO_2 permeability for the different MOFs/PIM-1 polymer-based MMMOFs reported in the literature (references are provided in Table S10).

MOFs	Polymer	Loading (wt.%)	Measurement conditions			CO_2 permeability (Barrer)			CO_2/CH_4 selectivity			*Ref.*
			P (bar)	method	*T* (ºC)	polymer	MMM$	Enh# (%)	polymer	MMM	Enh# (%)	
**AlFFIVE**-1-Ni	PIM-1	38	2.0	50/50	25	3796.3	5847.5	54.0	15.9	18.2	13.8	*This work*
UiO-66	PIM-1	28	5.0	single	25	4770.1	7610.1	59.5	16.6	14.4	-13.2	*(34)*
UiO-66-NH_2	PIM-1	20	4.0	single	25	3054.0	2210.0	-27.6	14.5	25.1	73.1	*(36)*
ZIF-8	PIM-1	43	1.0	single	22	4390.3	6300.0	43.5	14.2	14.6	2.8	*(37)*
MIL-101	PIM-1	47	1.0	single	25	5940.4	10600.0	78.5	11.0	10.9	-1.2	*(38)*

Table S10

A comparison of single and mixed CO_2/CH_4 separation performances, as well as selectivity (S) and permeability (P) enhancement of MOFs/PIM-1 MMMOF membrane.

MOFs	Polymer	Loading (wt%)	Measurement conditions			CO_2 permeability (barrer)	CO_2/CH_4 selectivity	Ref.
			P (bar)	method	*T* (°C)			
**AlFFIVE-1-Ni**	PIM-1	38.3	2.0	50/50	35	5847.5	18.2	*This work*
**AlFFIVE-1-Ni**	6FDA-DAM	59.2	2.0	50/50	35	1286.4	40.3	*(21)*
	6FDA-DAM-DAT	55.7				578.4	78.3	
Y-*fum*-fcu-MOF	6FDA-DAM	30.1	1.4	single	35	1365.0	23.3	*(63)*
	6FDA-DAM-DABA	17.7				286.8	38.0	
	6FDA-DAM	20.0				952.4	26.3	*(64)*
NbOFFIVE-1-Ni	6FDA-DAM	20.0	6.9	50/50	35	1007.8	26.3	*(17)*
MOF-74	6FDA-Durene	21.0	1.0	50/50	35	2250.0	15.7	*(65)*
	6FDA-DAT	15.0				52.0	55.6	
UiO-66	6FDA-ODA	25.0	10.0	single	35	50.4	46.1	*(66)*
	6FDA-ODA	25.0	10.0	50/50		54.4	42.3	
	PIM-1	28.6	5.0	50/50	25	7610.0	14.4	*(34)*
UiO-66-NH_2	6FDA-ODA	25.0	10.0	50/50	35	13.7	44.7	*(66)*
	PIM-1	20.0	4.0	single	25	2210.0	25.1	*(36)*
MIL-53	6FDA-DAM:HAB	15.0	10.0	single	35	71.1	15.1	*(67)*
	Matrimid®5218	15.0	3.0	single	25	12.4	51.8	*(67)*
	6FDA-ODA:DAM	20.0	10.0	50/50	35	61.5	13.0	*(68)*
MIL-53-NH_2	6FDA-DAM:HAB	20.0	10.0	pure	35	44.3	27.7	*(67)*
	6FDA-DAM	20.0	2.0	50/50	25	680.0	27.9	*(69)*
	6FDA-ODA:DAM	15.0	10.0	50/50	25	113.0	28.5	*(68)*
sod-ZMOF	6FDA-DAM	20.0	2.1	50/50	35	1408.3	26.4	*(70)*
ZIF-7	Pebax	34.0	3.7	single	20	41.0	44.0	*(71)*
ZIF-8	Matrimid®5218	30.0	5.0	50/50	35	25.1	53.0	*(72)*
	PIM-1	43.0	1.0	single	22	6300.0	14.6	*(37)*
	6FDA-Durene	41.9	10.0	single	35	779.0	20.9	*(73)*
ZIF-71	6FDA-Durene	20.0	3.5	50/50	35	3435.0	16.0	*(74)*
ZIF-90	6FDA-DAM	15.0	2.0	single	25	800.0	28.0	*(75)*

HKUST-1	Matrimid®5218	30.0	10.0	50/50	25	14.2	46.0	*(76)*
	ODPA-TMPDA	20.0	2.0	single	35	142.5	28.0	*(77)*
	6FDA-ODA	25.0	10.0	single	35	21.8	51.2	*(66)*
MOF-5	Matrimid®5218	30.0	2.0	single	35	20.2	44.7	*(78)*
MIL-101	PIM-1	47.0	1.0	single	25	10600	10.9	*(38)*

Table S11

Summary of representative MOFs MMMs for CO_2/CH_4 separation.

Supplementary Data S1

interface_MOF_6FDA-DAM.vesta

Simulated AlFFIVE-1-Ni/6FDA-DAM composite model.

Supplementary Data S2

interface_MOF_PIM-1.vesta

Simulated AlFFIVE-1-Ni/PIM-1 composite model.

REFERENCES AND NOTES

1. H. Dou, M. Xu, B. Wang, Z. Zhang, G. Wen, Y. Zheng, D. Luo, L. Zhao, A. Yu, L. Zhang, Microporous framework membranes for precise molecule/ion separations. *Chem. Soc. Rev.* **50**, 986–1029 (2021).

2. K. Chen, S. H. Mousavi, R. Singh, R. Q. Snurr, G. Li, P. A. Webley, Gating effect for gas adsorption in microporous materials-mechanisms and applications. *Chem. Soc. Rev.* **51**, 1139–1166 (2022).

3. G. Cai, P. Yan, L. Zhang, H.-C. Zhou, H.-L. Jiang, Metal-organic framework-based hierarchically porous materials: Synthesis and applications. *Chem. Rev.* **121**, 12278–12326 (2021).

4. D. S. Sholl, R. P. Lively, Seven chemical separations to change the world. *Nature* **532**, 435–437 (2016).

5. M. Kalaj, K. C. Bentz, S. Ayala Jr., J. M. Palomba, K. S. Barcus, Y. Katayama, S. M. Cohen, Mof-polymer hybrid materials: From simple composites to tailored architectures. *Chem. Rev.* **120**, 8267–8302 (2020).

6. L. M. Robeson, The upper bound revisited. *J. Membr. Sci.* **320** 390–400 (2008).

7. R. T. Adams, J. S. Lee, T.-H. Bae, J. K. Ward, J. Johnson, C. W. Jones, S. Nair, W. J. Koros, CO_2-CH_4 permeation in high zeolite 4A loading mixed matrix membranes. *J. Membr. Sci.* **367**, 197–203 (2011).

8. T. Rodenas, I. Luz, G. Prieto, B. Seoane, H. Miro, A. Corma, F. Kapteijn, F. X. Llabrés i Xamena, J. Gascon, Metal-organic framework nanosheets in polymer composite materials for gas separation. *Nat. Mater.* **14**, 48–55 (2015).

9. A. Pustovarenko, M. G. Goesten, S. Sachdeva, M. Shan, Z. Amghouz, Y. Belmabkhout, A. Dikhtiarenko, T. Rodenas, D. Keskin, I. K. Voets, B. M. Weckhuysen, M. Eddaoudi, L. C. P. M. de Smet, E. J. R. Sudhölter, F. Kapteijn, B. Seoane, J. Gascon, Nanosheets of nonlayered

aluminum metal-organic frameworks through a surfactant-assisted method. *Adv. Mater.* **30**, e1707234 (2018).

10. Y.-Z. Li, Z.-H. Fu, G. Xu, Metal-organic framework nanosheets: Preparation and applications. *Coord. Chem. Rev.* **388**, 79–106 (2019).

11. M. Galizia, W. S. Chi, Z. P. Smith, T. C. Merkel, R. W. Baker, B. D. Freeman, 50th anniversary perspective: Polymers and mixed matrix membranes for gas and vapor separation: A review and prospective opportunities. *Macromolecules* **50**, 7809–7843 (2017).

12. X. Qian, M. Ostwal, A. Asatekin, G. M. Geise, Z. P. Smith, W. A. Phillip, R. P. Lively, J. R. McCutcheon, A critical review and commentary on recent progress of additive manufacturing and its impact on membrane technology. *J. Membr. Sci.* **645**, 120041 (2022).

13. Q. Qian, P. A. Asinger, M. J. Lee, G. Han, K. Mizrahi Rodriguez, S. Lin, F. M. Benedetti, A. X. Wu, W. S. Chi, Z. P. Smith, MOF-based membranes for gas separations. *Chem. Rev.* **120**, 8161–8266 (2020).

14. Y. Liu, H. Wu, S. Wu, S. Song, Z. Guo, Y. Ren, R. Zhao, L. Yang, Y. Wu, Z. Jiang, Multifunctional covalent organic framework (COF)-based mixed matrix membranes for enhanced CO_2 separation. *J. Membr. Sci.* **618**, 118693 (2021).

15. Q. Zhang, S. Li, C. Wang, H.-C. Chang, R. Guo, Carbon nanotube-based mixed-matrix membranes with supramolecularly engineered interface for enhanced gas separation performance. *J. Membr. Sci.* **598**, 117794 (2020).

16. S. J. Datta, C. Khumnoon, Z. H. Lee, W. K. Moon, S. Docao, T. H. Nguyen, I. C. Hwang, D. Moon, P. Oleynikov, O. Terasaki, CO_2 capture from humid flue gases and humid atmosphere using a microporous coppersilicate. *Science* **350**, 302–306 (2015).

7. G. Liu, A. Cadiau, Y. Liu, K. Adil, V. Chernikova, I. D. Carja, Y. Belmabkhout, M. Karunakaran, O. Shekhah, C. Zhang, Enabling fluorinated MOF-based membranes for simultaneous removal of H_2S And CO_2 from natural gas. *Angew Chem Int Ed Engl* **130**, 15027–15032 (2018).

18. K. Adil, Y. Belmabkhout, R. S. Pillai, A. Cadiau, P. M. Bhatt, A. H. Assen, G. Maurin, M. Eddaoudi, Gas/vapour separation using ultra-microporous metal–organic frameworks: Insights into the structure/separation relationship. *Chem. Soc. Rev.* **46**, 3402–3430 (2017).

19. M. Shete, P. Kumar, J. E. Bachman, X. Ma, Z. P. Smith, W. Xu, K. A. Mkhoyan, J. R. Long, M. Tsapatsis, On the direct synthesis of Cu(BDC) MOF nanosheets and their performance in mixed matrix membranes. *J. Membr. Sci.* **549**, 312–320 (2018).

20. X. Bi, Y. Zhang, F. Zhang, S. Zhang, Z. Wang, J. Jin, MOF nanosheet-based mixed matrix membranes with metal-organic coordination interfacial interaction for gas separation. *ACS Appl. Mater. Interfaces* **12**, 49101–49110 (2020).

21. S. J. Datta, A. Mayoral, N. Murthy Srivatsa Bettahalli, P. M. Bhatt, M. Karunakaran, I. D. Carja, D. Fan, P. G. M Mileo, R. Semino, G. Maurin, O. Terasaki, M. Eddaoudi, Rational design of mixed-matrix metal-organic framework membranes for molecular separations. *Science* **376**, 1080–1087 (2022).

22. C. Y. Chuah, K. Goh, Y. Yang, H. Gong, W. Li, H. E. Karahan, M. D. Guiver, R. Wang, T.-H. Bae, Harnessing filler materials for enhancing biogas separation membranes. *Chem. Rev.* **118**, 8655–8769 (2018).

23. H. B. Park, J. Kamcev, L. M. Robeson, M. Elimelech, B. D. Freeman, Maximizing the right stuff: The trade-off between membrane permeability and selectivity. *Science* **356**, eaab0530 (2017).

24. Y. Peng, Y. Li, Y. Ban, H. Jin, W. Jiao, X. Liu, W. Yang, Metal-organic framework nanosheets as building blocks for molecular sieving membranes. *Science* **346**, 1356–1359 (2014).

25. A. Cadiau, Y. Belmabkhout, K. Adil, P. M. Bhatt, R. S. Pillai, A. Shkurenko, C. Martineau-Corcos, G. Maurin, M. Eddaoudi, Hydrolytically stable fluorinated metal-organic frameworks for energy-efficient dehydration. *Science* **356**, 731–735 (2017).

26. Y. Belmabkhout, P. M. Bhatt, K. Adil, R. S. Pillai, A. Cadiau, A. Shkurenko, G. Maurin, G. Liu, W. J. Koros, M. Eddaoudi, Natural gas upgrading using a fluorinated MOF with tuned H_2S and CO_2 adsorption selectivity. *Nat. Energy* **3**, 1059–1066 (2018).

27. M. Tchalala, P. Bhatt, K. Chappanda, S. Tavares, K. Adil, Y. Belmabkhout, A. Shkurenko, A. Cadiau, N. Heymans, G. De Weireld, Fluorinated MOF platform for selective removal and sensing of SO_2 from flue gas and air. *Nat. Commun.* **10**, 1328 (2019).

28. R. Semino, N. A. Ramsahye, A. Ghoufi, G. Maurin, Microscopic model of the metal–organic framework/polymer interface: A first step toward understanding the compatibility in mixed matrix membranes. *ACS Appl. Mater. Interfaces* **8**, 809–819 (2016).

29. A. Ozcan, R. Semino, G. Maurin, A. O. Yazaydin, Modeling of gas transport through polymer/mof interfaces: A microsecond-scale concentration gradient-driven molecular dynamics study. *Chem. Mater.* **32** 1288–1296 (2020).

30. D. Fan, A. Ozcan, N. A. Ramsahye, D. Zhao, G. Maurin, R. Semino, Is porosity at the MOF/polymer interface necessarily an obstacle to optimal gas-separation performances in mixed matrix membranes? *ACS Mater. Lett.* **3**, 344–350 (2021).

31. A. Ozcan, C. Perego, M. Salvalaglio, M. Parrinello, O. Yazaydin, Concentration gradient driven molecular dynamics: A new method for simulations of membrane permeation and separation. *Chem. Sci.* **8**, 3858–3865 (2017).

32. J. Kärger, D. M. Ruthven, D. N. Theodorou, *Diffusion In Nanoporous Materials* (Wiley Online Library, 2012), vol. 48.

33. H. J. Bakker, J. L. Skinner, Vibrational spectroscopy as a probe of structure and dynamics in liquid water. *Chem. Rev.* **110**, 1498–1517 (2010).

34. M. R. Khdhayyer, E. Esposito, A. Fuoco, M. Monteleone, L. Giorno, J. C. Jansen, M. P. Attfield,P. M. Budd, Mixed matrix membranes based on UiO-66 MOFs in the polymer of intrinsic microporosity PIM-1. *Sep. Purif. Technol.* **173**, 304–313 (2017).

35. I.-D. Carja, S. R. Tavares, O. Shekhah, A. Ozcan, R. Semino, V. S. Kale, M. Eddaoudi, G. Maurin, Insights into the enhancement of MOF/polymer adhesion in mixed-matrix membranes via polymer functionalization. *ACS Appl. Mater. Interfaces* **13**, 29041–29047 (2021).

36. B. Ghalei, K. Sakurai, Y. Kinoshita, K. Wakimoto, A. P. Isfahani, Q. Song, K. Doitomi, S. Furukawa, H. Hirao, H. Kusuda, S. Kitagawa, E. Sivaniah, Enhanced selectivity in mixed matrix membranes for CO_2 capture through efficient dispersion of amine-functionalized MOF nanoparticles. *Nat. Energy* **2**, 1–9 (2017).

37. A. F. Bushell, M. P. Attfield, C. R. Mason, P. M. Budd, Y. Yampolskii, L. Starannikova, A. Rebrov, F. Bazzarelli, P. Bernardo, J. C. Jansen, M. Lanč, K. Friess, V. Shantarovich, V. Gustov, V. Isaeva, Gas permeation parameters of mixed matrix membranes based on the polymer of intrinsic microporosity PIM-1 and the zeolitic imidazolate framework ZIF-8. *J. Membr. Sci.* **427** 48–62 (2013).

38. M. Khdhayyer, A. F. Bushell, P. M. Budd, M. P. Attfield, D. Jiang, A. D. Burrows, E. Esposito, P. Bernardo, M. Monteleone, A. Fuoco, G. Clarizia, F. Bazzarelli, A. Gordano, J. C. Jansen, Mixed matrix membranes based on MIL-101 metal–organic frameworks in polymer of intrinsic microporosity PIM-1. *Sep. Purif. Technol.* **212** 545–554 (2019).

39. J. P. Perdew, K. Burke, M. Ernzerhof, Generalized gradient approximation made simple. *Phys. Rev. Lett.* **77**, 3865–3868 (1996).

40. G. Kresse, J. Furthmüller, Efficient iterative schemes for *ab initio* total-energy calculations using a plane-wave basis set. *Phys. Rev. B* **54**, 11169–11186 (1996).

41. S. Plimpton, P. Crozier, A. Thompson, LAMMPS-large-scale atomic/molecular massively parallel simulator. *Sandia Natl. Lab.* **18**, 43 (2007).

42. Lammps Molecular Dynamics Simulator, https://lammps.org/#gsc.tab=0.

43. A. K. Rappé, C. J. Casewit, K. Colwell, W. A. Goddard III, W. M. Skiff, UFF, a full periodic table force field for molecular mechanics and molecular dynamics simulations. *J. Am. Chem. Soc.* **114**, 10024–10035 (1992).

44. S. L. Mayo, B. D. Olafson, W. A. Goddard, DREIDING: A generic force field for molecular simulations. *J. Phys. Chem.* **94**, 8897–8909 (1990).

45. J. Wang, R. M. Wolf, J. W. Caldwell, P. A. Kollman, D. A. Case, Development and testing of a general amber force field. *J. Comput. Chem.* **25**, 1157–1174 (2004).

46. J. G. Harris, K. H. Yung, Carbon dioxide's liquid-vapor coexistence curve and critical properties as predicted by a simple molecular model. *J. Phys. Chem.* **99**, 12021–12024 (1995).

47. M. J. Abraham, T. Murtola, R. Schulz, S. Páll, J. C. Smith, B. Hess, E. Lindahl, GROMACS: High performance molecular simulations through multi-level parallelism from laptops to supercomputers. *SoftwareX*, **1-2** 19–25 (2015).

48. G. A. Tribello, M. Bonomi, D. Branduardi, C. Camilloni, G. Bussi, PLUMED 2: New feathers for an old bird. *Comput. Phys. Commun.* **185**, 604–613 (2014).

49. C. F. Abrams, E. Vanden-Eijnden, Large-scale conformational sampling of proteins using temperature-accelerated molecular dynamics. *Biophys. J.* **98**, 26a (2010).

50. M. R. So rensen, A. F. Voter, Temperature-accelerated dynamics for simulation of infrequent events. *J. Chem. Phys.* **112**, 9599–9606 (2000).

51. S. Nosé, A molecular dynamics method for simulations in the canonical ensemble. *Mol. Phys.* **52**, 255–268 (1984).

52. U. Essmann, L. Perera, M. L. Berkowitz, T. Darden, H. Lee, L. G. Pedersen, A smooth particle mesh ewald method. *J. Chem. Phys.* **103**, 8577–8593 (1995).

53. S. Grimme, Semiempirical GGA-type density functional constructed with a long-range dispersion correction. *J. Comput. Chem.* **27**, 1787–1799 (2006).

54. S. Dudarev, G. Botton, S. Savrasov, C. Humphreys, A. Sutton, Electron-energy-loss spectra and the structural stability of nickel oxide: An LSDA+ U study. *Phys. Rev. B* **57**, 1505 (1998).

55. T. A. Manz, N. G. Limas, Introducing DDEC6 atomic population analysis: Part 1. Charge partitioning theory and methodology. *RSC Adv.* **6**, 47771–47801 (2016).

56. M. Z. Ahmad, M. Navarro, M. Lhotka, B. Zornoza, C. Téllez, W. M. de Vos, N. E. Benes, N. M. Konnertz, T. Visser, R. Semino, G. Maurin, V. Fila, J. Coronas, Enhanced gas separation performance of 6FDA-DAM based mixed matrix membranes by incorporating MOF UiO-66 and its derivatives. *J. Membr. Sci.* **558**, 64–77 (2018).

57. L. J. Abbott, K. E. Hart, C. M. Colina, *Polymatic*: A generalized simulated polymerization algorithm for amorphous polymers. *Theor. Chem. Acc.* **132**, 1334 (2013).

58. R. Semino, J. P. Dürholt, R. Schmid, G. Maurin, Multiscale modeling of the HKUST-1/poly (vinyl alcohol) interface: From an atomistic to a coarse graining approach. *J. Phys. Chem. C* **121**, 21491–21496 (2017).

59. H. J. C. Berendsen, J. P. M. Postma, W. F. van Gunsteren, A. DiNola, J. R. Haak, Molecular dynamics with coupling to an external bath. *J. Chem. Phys.* **81**, 3684–3690 (1984).

60. I. T. Todorov, W. Smith, K. Trachenko, M. T. Dove, DL_POLY_3: New dimensions in molecular dynamics simulations via massive parallelism. *J. Mater. Chem.* **16**, 1911–1918 (2006).

61. H. A. Lorentz, (1881) Ueber die Anwendung des Satzes vom Virial in der kinetischen Theorie der Gase. *Ann. Phys.* **248**, 127–136 (1881).

62. P. P. Ewald, Die Berechnung optischer und elektrostatischer Gitterpotentiale. *Ann. Phys.* **369**, 253–287 (1921).

63. G. Liu, V. Chernikova, Y. Liu,K. Zhang, Y. Belmabkhout, O. Shekhah, C. Zhang, S. Yi, M. Eddaoudi, W. J. Koros, Mixed matrix formulations with MOF molecular sieving for key energy-intensive separations. *Nat. Mater.* **17**, 283–289 (2018).

64. Y. Liu, G. Liu, C. Zhang, W. Qiu, S. Yi, V. Chernikova, Z. Chen, Y. Belmabkhout, O. Shekhah, M. Eddaoudi, Enhanced CO_2/CH_4 separation performance of a mixed matrix membrane based on tailored MOF-polymer formulations. *Adv. Sci.* **5**, 1800982 (2018).

65. J. E. Bachman, J. R. Long, Plasticization-resistant Ni_2(dobdc)/polyimide composite membranes for the removal of CO_2 from natural gas. *Energ. Environ. Sci.* **9**, 2031–2036 (2016).

66. O. G. Nik, X. Y. Chen, S. Kaliaguine, Functionalized metal organic framework-polyimide mixed matrix membranes for CO_2/CH_4 separation. *J. Membr. Sci.* **413-414**, 48–61 (2012).

67. N. Tien-Binh, H. Vinh-Thang, X. Y. Chen, D. Rodrigue, S. Kaliaguine, Polymer functionalization to enhance interface quality of mixed matrix membranes for high CO_2/CH_4 gas separation. *J. Mater. Chem. A* **3**, 15202–15213 (2015).

68. X. Y. Chen, V.-T. Hoang, D. Rodrigue, S. Kaliaguine, Optimization of continuous phase in amino-functionalized metal-organic framework (MIL-53) based co-polyimide mixed matrix membranes for CO_2/CH_4 separation. *RSC Adv.* **3**, 24266–24279 (2013).

69. A. Sabetghadam, B. Seoane, D. Keskin, N. Duim, T. Rodenas, S. Shahid, S. Sorribas, C. L. Guillouzer, G. Clet, C. Tellez, M. Daturi, J. Coronas, F. Kapteijn, J. Gascon, Metal organic framework crystals in mixed-matrix membranes: Impact of the filler morphology on the gas separation performance. *Adv. Funct. Mater.* **26**, 3154–3163 (2016).

70. G. Liu, Y. Labreche, V. Chernikova, O. Shekhah, C. Zhang, Y. Belmabkhout, M. Eddaoudi, W. J. Koros, Zeolite-like MOF nanocrystals incorporated 6FDA-polyimide mixed-matrix membranes for CO_2/CH_4 separation. *J. Membr. Sci.* **565**, 186–193 (2018).

71. T. Li,Y. Pan, K.-V. Peinemann, Z. Lai, Carbon dioxide selective mixed matrix composite membrane containing ZIF-7 nano-fillers. *J. Membr. Sci.* **425-426**, 235–242 (2013).

72. S. Shahid, K. Nijmeijer, S. Nehache, I. Vankelecom, A. Deratani, D. Quemener, MOF-mixed matrix membranes: Precise dispersion of MOF particles with better compatibility via a particle fusion approach for enhanced gas separation properties. *J. Membr. Sci.* **492**, 21–31 (2015).

73. M. Askari, T.-S. Chung, Natural gas purification and olefin/paraffin separation using thermal cross-linkable co-polyimide/ZIF-8 mixed matrix membranes. *J. Membr. Sci.* **444**, 173–183 (2013).

74. S. Japip, H. Wang, Y. Xiao, T. S. Chung, Highly permeable zeolitic imidazolate framework (ZIF)-71 nano-particles enhanced polyimide membranes for gas separation. *J. Membr. Sci.* **467**, 162–174 (2014).

75. T. H. Bae, J. S. Lee, W. Qiu, W. J. Koros, C. W. Jones, S. Nair, A high-performance gas-separation membrane containing submicrometer-sized metal-organic framework crystals. *Angew. Chem. Int. Ed. Engl.* **122**, 10059–10062 (2010).

76. S. Shahid, K. Nijmeijer, Performance and plasticization behavior of polymer-MOF membranes for gas separation at elevated pressures. *J. Membr. Sci.* **470**, 166–177 (2014).

77. C. Duan, X. Jie, D. Liu, Y. Cao, Q. Yuan, Post-treatment effect on gas separation property of mixed matrix membranes containing metal organic frameworks. *J. Membr. Sci.*, **466**, 92–102 (2014).

78. E. V. Perez, K. J. Balkus Jr., J. P. Ferraris, I. H. Musselman, Mixed-matrix membranes containing MOF-5 for gas separations. *J. Membr. Sci.* **328**, 165–173 (2009).
